# Famine Foods: A Structured Narrative Review of Nutritional, Toxicological, Ecological, and Socio-Cultural Perspectives

**DOI:** 10.3390/foods15203602

**Published:** 2026-10-10

**Authors:** José Luis Guil Guerrero, Jesús Enrique de las Heras-Roger, Miguel Ángel Rincón-Cervera, Carlos Díaz-Romero

**Affiliations:** 1 Department of Agronomy, Food Technology Area, University of Almería, 04120 La Cañada, Spain; marincer@inta.uchile.cl; 2 Department of Chemical Engineering and Pharmaceutical Technology, University of La Laguna, 38200 Santa Cruz de Tenerife, Spain; jherasro@ull.edu.es (J.E.d.l.H.-R.); cdiaz@ull.edu.es (C.D.-R.); 3 Cátedra de Agroturismo y Enoturismo de Canarias, Instituto Canario de Calidad Agroalimentaria (ICCA-ULL), 38200 San Cristóbal de La Laguna, Spain; 4 Institute of Nutrition and Food Technology, University of Chile, Macul 5524, Santiago, Chile

**Keywords:** wild edible plants (WEPs), neglected and underutilised species (NUS), food security, traditional ecological knowledge (TEK), agroecology, survival strategies

## Abstract

Famine foods are wild, neglected, or unconventional biological resources consumed during periods of food insecurity, agricultural failure, inter-harvest periods, and socio-political disruption. This review examines famine foods through an interdisciplinary perspective integrating nutrition, toxicology, ethnobiology, ecology, history, and food security studies. Botanical and zoological famine resources, including wild edible plants, algae, insects, molluscs, rodents, roots, bark tissues, and emergency animal products, are evaluated regarding their nutritional composition, toxicological risks, and cultural significance. Many famine foods contain substantial amounts of micronutrients, essential fatty acids, bioactive compounds, and carbohydrates that historically contributed to survival during famine conditions. However, their utilisation is limited by antinutritional factors, toxic secondary metabolites, zoonotic hazards, and the need for labour-intensive detoxification methods grounded in Traditional Ecological Knowledge. Socio-cultural stigma associated with famine foods, their role in resilience and oral history, and the influence of political and economic systems on food access during crises are also analysed. Environmental perspectives highlight biodiversity as a critical buffer against crop failure, while climate variability increasingly intensifies dependence on wild resources. Contemporary interest in neglected and underutilised species suggests that some famine foods may contribute to sustainable and climate-resilient food systems. Nevertheless, important gaps remain regarding toxicological standardisation, nutrient bioavailability and conservation.

## 1. Introduction

The modern concept of food security, formalised during the 1996 World Food Summit, is commonly structured around four interrelated dimensions: food availability, access, utilisation, and stability [1]. Within this framework, food security is generally understood as a stable condition in which populations maintain reliable access to sufficient, safe, and nutritious food. Famine is the most extreme expression of food insecurity and reveals the fragility of our food system.

Famines are complex socio-ecological crises that emerge through the interaction of environmental stress, agricultural disruption, economic instability, and unequal access to food resources [2,3]. Although climatic events such as droughts, floods, and pest outbreaks may reduce food availability, famine severity is often shaped by political, economic, and institutional failures that constrain access to food among vulnerable populations [4]. Historical evidence from the Great Famine of 1696–1697 in Northern Europe, the Irish Potato Famine, the Ukrainian Holodomor (1932–1933), the siege of Sarajevo during the Bosnian War (1992–1995), and more recent humanitarian crises such as the Syrian conflict demonstrates that famine rarely results from food shortage alone. Instead, it commonly reflects the interaction between environmental stress and political, economic, or military disruptions that severely restrict access to food resources [5,6,7,8,9,10].

During acute food shortages, conventional food systems often fail to meet basic nutritional needs. Under such conditions, households frequently adopt alternative survival strategies—also referred to as coping mechanisms—to compensate for declining food access and income [11]. These strategies vary in severity and may include reducing meal frequency, selling productive assets, migrating, relying on social networks, and consuming famine foods. This consumption represents one of the more severe adaptive responses, typically indicating significant deterioration in food access and livelihood stability [12,13].

The concept of famine foods sits at an important intersection between institutional models of food security and localised survival strategies. Conventional food-security frameworks typically evaluate hunger through macro-level indicators such as food availability, economic access, nutritional adequacy, and system stability. However, during severe scarcity, affected populations often rely on informal, adaptive practices that fall outside formal agricultural and market systems. Among these practices is the consumption of famine foods, which can be considered unconventional biological resources utilised when preferred foods become inaccessible or unavailable. A key distinction exists between conventional staple foods and famine food substitutes. Staple crops such as rice, maize, wheat, and potatoes have undergone extensive domestication and selection for high productivity, digestibility, storage stability, and caloric efficiency [14]. However, these systems frequently depend on stable climatic conditions, organised agricultural infrastructure, and functioning markets. By contrast, many famine foods are derived from wild or semi-wild species adapted to marginal environments and ecological disturbance. Their persistence under drought, flooding, or pest pressure makes them important fall-back resources when conventional agricultural systems fail [15]. The persistence of these practices across historical and contemporary famines underscores that famine foods should be understood not merely as relics of past deprivation, but as enduring components of human adaptive capacity under environmental uncertainty, armed conflict, and systemic food insecurity.

State-centred food-security frameworks often prioritise restoring markets and conventional agriculture, whereas local survival strategies emphasise ecological diversification and adaptive resource use. Consequently, famine foods should not be viewed exclusively as evidence of systemic collapse, but also as components of localised resilience strategies and active adaptive responses shaped by ecological knowledge, cultural practices, and survival priorities. This perspective complicates the distinction between “food insecurity” and “resilience” [16,17].

Famine foods may include many unconventional food groups, such as wild plants, unconventional animal species (including large insects), agricultural by-products, mushrooms, and processed substitutes. They are consumed during crises temporarily to maintain a minimal caloric intake [18]. Although frequently stigmatised under normal conditions, famine foods often function as critical buffers against starvation during environmental, economic, or political disruptions. Despite their historical significance, famine foods remain comparatively understudied within mainstream nutrition, agronomy, and public health research. Existing scholarship is fragmented across disciplines, including anthropology, toxicology, ecology, ethnobiology, and food security studies [19].

Consequently, comprehensive interdisciplinary syntheses remain limited. New strategies are therefore needed to integrate nutritional, ecological, anthropological, and socio-political perspectives. These practices are often interpreted as indicators of extreme deprivation, but recent ethnobotanical research suggests a more nuanced interpretation [20,21]. At the same time, growing concerns regarding climate change, biodiversity loss, food-system vulnerability, and nutritional insecurity have renewed scientific interest in neglected and underutilised food resources [22].

Importantly, studying famine foods highlights the limitations of narrowly production-oriented models of food security. While institutional frameworks tend to emphasise agricultural productivity and market integration, famine-food practices demonstrate how communities mobilise traditional ecological knowledge (TEK), biodiversity and adaptive flexibility within vulnerable livelihood systems [23] and to navigate periods of systemic instability. TEK refers to the cumulative body of ecological knowledge, practices, and beliefs developed through long-term interaction between communities and local environments [24]. Communities relying on these resources often possess detailed knowledge regarding species identification, seasonal availability, harvesting methods, and detoxification practices. Ethnobotanical and anthropological studies have demonstrated that TEK plays a central role in identifying, harvesting, processing, preparing, detoxifying and safely consuming many emergency food resources [15].

### 1.1. Classification of Famine Foods

Famine foods refer to a broad range of uncultivated biological materials and other locally available food, consumed as alternative or supplementary resources when conventional food access is substantially disrupted by severe scarcity, agricultural failure, conflict, displacement, or related crises. Their classification therefore depends primarily on the context and function of consumption rather than on the biological identity of the resource [25,26]. They are broadly classified into three categories according to their biological origin, processing requirements, and ecological accessibility: (1) wild plants; (2) unconventional animal resources; and (3) processed substitutes (Table 1). Although these categories overlap in practice, they provide a useful framework for understanding the diversity of emergency food systems and the adaptive strategies associated with them. The same species may constitute a traditional food in one cultural context, a fallback food in another, or a famine food when consumed specifically under conditions of severe food scarcity.

#### 1.1.1. Wild Edible Plants (WEPs)

WEPs constitute the most widely documented category of famine foods. These resources include uncultivated leaves, roots, tubers, seeds, fruits, lichens, and tree bark, which are harvested from forests, grasslands, and marginal environments during periods of scarcity [23]. In many regions, drought-resistant or perennial species become particularly important when cultivated crops fail. For example, communities in Eastern Ethiopia rely on species such as *Balanites aegyptiaca*, *Dioscorea* spp., and *Moringa stenopetala* during recurrent droughts and seasonal food shortages [31,32].

Historical evidence also demonstrates the use of highly unconventional plant materials under severe famine conditions. In northern Europe, populations consumed bread prepared from pine bark flour and lichens to extend diminishing grain supplies [13]. Although these resources often provide limited caloric value and may contain antinutritional compounds, they contribute to essential micronutrient intake and, in the short term, to dietary bulk.

#### 1.1.2. Unconventional Animals and Mini-Livestock

Animal-based famine foods typically emerge when conventional livestock becomes unavailable, economically inaccessible, or too valuable to slaughter. Under such circumstances, communities may rely on unconventional and/or wild animals, such as rodents, rabbits, insects, spiders, molluscs, amphibians, and other abundant fauna [28]. Research has drawn attention to the nutritional and ecological importance of arthropods as emergency protein sources due to their relatively high protein density, efficient reproduction, and low environmental requirements [29].

However, the classification of these organisms as “famine foods” varies culturally. In some societies, insects and small vertebrates are integrated into traditional diets outside crisis periods, whereas in others they are strongly stigmatised and consumed only under severe scarcity. This distinction highlights the importance of cultural context in defining what constitutes an emergency food resource [33].

#### 1.1.3. Processed Substitutes and Technological Adaptations

A third category includes processed substitutes derived from materials that are either marginally edible or normally excluded from human diets. These foods typically require labour-intensive preparation methods to improve digestibility, reduce toxicity, or increase volume. Historical examples include acorn flour bread, bark flour mixtures, famine gruels, seaweed preparations, and bone-derived food additives [13].

Contemporary discussions of famine resilience have expanded this category to include technologically produced emergency foods such as single-cell proteins, microbial biomass, and industrial seaweed systems designed for catastrophic food-system disruptions [30]. Although these innovations differ fundamentally from foods traditionally used during famine periods, both represent adaptive attempts to secure nutrition amid systemic instability.

### 1.2. Cultural Perceptions of Famine Foods

The distinction between “food” and “non-food” is not solely biological, but also cultural and symbolic. Famine foods often occupy an ambiguous social position because people consume them outside culturally accepted dietary norms. Anthropological studies have shown that food functions not only as a source of energy and nutrients, but also as a marker of identity, dignity, social belonging, and cultural continuity [34]. Consequently, consuming famine foods may carry significant social and psychological implications beyond their nutritional value (Table 2).

In many societies, emergency foods are viewed as temporary “bridge foods” consumed only during crises. Lasco and Mendoza [34], for example, described the Filipino concept of *pantawid-gutom* (“to bridge hunger”), referring to substances consumed to temporarily suppress hunger rather than provide socially recognised meals. Similar distinctions appear historically in European famine contexts, where bark bread and lichen-based foods were often regarded as symbols of deprivation rather than legitimate dietary alternatives [13].

The use of famine foods may therefore generate social stigma, particularly when these resources are associated with poverty, marginalisation, or social exclusion. Historical and ethnographic accounts suggest that severe food scarcity can also disrupt social cohesion and traditional food-sharing practices. Under extreme conditions, individuals may conceal access to food resources or consume stigmatised materials privately to avoid shame, theft, or social judgment [35].

At the same time, cultural perceptions of famine foods are dynamic rather than fixed. Resources once associated with deprivation may later be re-evaluated as heritage foods, nutritionally valuable wild species, or sustainable alternatives within modern food systems. This transition illustrates how social meanings surrounding food are shaped by historical context, economic conditions, and changing environmental priorities [27].

### 1.3. Objectives of the Review

The use of famine foods reflects the adaptive capacity of human populations to exploit ecological diversity under conditions of extreme nutritional stress. Some foods used in famine periods may contain toxic compounds and toxins capable of causing severe health consequences if improperly processed [36]. Therefore, in many cases, famine foods require extensive preparation before consumption to eliminate these toxic or antinutritional compounds. Traditional processing techniques represent critical cultural adaptations that enable the safe consumption of otherwise hazardous unconventional foods.

This review aims to synthesise current knowledge on famine foods from nutritional, toxicological, ecological, and socio-cultural perspectives. Specifically, the present article examines the diversity and nutritional composition of plant- and animal-based famine foods; evaluates traditional processing strategies used to reduce toxicity; and explores the cultural perceptions and stigmatisation associated with their consumption. It also considers the contemporary relevance of famine foods in discussions of food-system resilience, biodiversity conservation, and sustainable adaptation to climate variability. By integrating evidence across disciplines, the review seeks to reposition famine foods, not only as indicators of historical deprivation, but also as important reservoirs of TEK and adaptive survival strategies.

## 2. Materials and Methods

This manuscript used a structured narrative review approach to identify, organise, and critically synthesise evidence on famine foods across historical, nutritional, toxicological, ethnobiological, ecological, and socio-cultural contexts. The methodological framework drew on established approaches to narrative synthesis and principles of transparent evidence mapping [37,38]. We selected literature based on its relevance to historical famine experiences, plant- and animal-based food resources, nutritional composition, traditional processing and detoxification practices, cultural perceptions, biodiversity, and contemporary food-system resilience.

The review was not designed as a systematic review and does not claim adherence to PRISMA reporting requirements. Instead, it used a structured search and screening procedure to improve traceability while retaining the flexibility required for multidisciplinary narrative synthesis.

### 2.1. Literature Search Strategy

We conducted a structured and targeted literature search across major scientific and historical databases, including PubMed, Scopus, ScienceDirect, SpringerLink, and Google Scholar. We selected these platforms to capture literature spanning food science, human nutrition, ethnobotany, history, political economy, and environmental sciences.

We used a targeted keyword strategy to identify literature addressing famine foods and their nutritional, toxicological, ethnobiological, ecological, and socio-cultural dimensions. The search combined key concepts using Boolean operators (AND, OR). Core search terms include “famine foods”, “nutritional value”, “wild edible plants”, “toxicity”, “neglected and underutilised species”, “food security”, “traditional ecological knowledge”, and “survival diets”. We added additional terms, such as “entitlement failure”, “emergency substitutes”, and “biocultural resilience”, to capture studies addressing adaptive responses to food scarcity. We adapted search strings to each database’s syntax requirements while preserving the same conceptual framework.

In addition to electronic database searches, we conducted backward citation tracking by screening the reference lists of seminal monographs and key reviews to identify foundational data not captured through standard keyword searches.

Additionally, we manually searched previous reviews and the reference lists of included studies and added relevant articles accordingly. We used Covidence to manage abstract and full-text screening and data extraction [39].

### 2.2. Eligibility Criteria and Study Selection

The eligibility criteria were structured according to the scope and objectives of the structured narrative review rather than a formal PICO framework. We considered studies and authoritative sources that addressed one or more of the principal evidence domains examined in this review. These domains included: (i) biological resources used as famine or fallback foods; (ii) nutritional composition and dietary relevance; (iii) toxic compounds, antinutritional factors, and processing or detoxification practices; (iv) Traditional Ecological Knowledge and cultural perceptions; (v) historical and socio-political determinants of food access; and (vi) ecological and contemporary food-system relevance.

Sources were considered eligible when they provided substantive information relevant to one or more of these domains. The review included studies addressing plant- and animal-based foods, wild and underutilised biological resources, traditional or emergency food practices, nutritional and biochemical characteristics, toxicological or antinutritional concerns, and the cultural, historical, ecological, or socio-economic contexts associated with their use. We considered historical accounts, ethnobiological studies, analytical investigations, reviews, and other authoritative sources when they contributed relevant evidence to the review objectives.

Sources were excluded when they lacked sufficient information to support the statements for which they were considered, or when their content was unrelated to famine foods, food scarcity, fallback resources, or the nutritional, toxicological, cultural, historical, ecological, or food-system dimensions addressed in this review. When several sources addressed the same topic, we prioritised primary studies, authoritative historical or ethnographic sources, and recent reviews with substantial evidence. We also used backward citation tracking to identify relevant seminal or otherwise informative sources not retrieved through the database searches.

Because the review integrates heterogeneous evidence rather than intervention-based studies, it did not impose a uniform intervention, comparator, or outcome structure. This approach allowed inclusion of evidence spanning different historical periods, geographical regions, biological resources, and methodological traditions while remaining aligned with the review’s predefined thematic scope. Information not reported by the original source was not inferred. Instead, missing information was treated as not reported and was considered accordingly during the narrative synthesis.

### 2.3. Inclusion and Exclusion Criteria

Only peer-reviewed articles, academic book chapters, and authoritative historical reports published in English were considered. Studies that focus explicitly on famine foods or emergency survival resources were required, and they report on at least one of the following aspects:

1. Biochemical Characterisation: Detailed profiling of macronutrients, micronutrients, toxins and anti-nutritional factors.
2. Ethnobotanical Documentation: Indigenous knowledge regarding the identification, harvesting, and detoxification of wild resources.
3. Historical and Political Analysis: Case studies of specific famine events linked to policy failure, conflict, or environmental collapse.
4. Modern Agronomic Relevance: The transition of famine resources into sustainable “future foods” or alternative food systems.

While priority was given to studies published from 1980 onward—coinciding with the emergence of modern entitlement theory and advanced chromatographic analysis—seminal historical records and foundational ethnobotanical texts from earlier eras were selectively included to provide essential diachronic context.

Exclusion criteria comprised non-peer-reviewed popular media, sources lacking sufficient information to support the statements for which they were considered, and publications focusing exclusively on primary commercial staples without reference to emergency substitution.

### 2.4. Data Extraction and Synthesis

We extracted and organised relevant information according to the principal evidence domains examined in this review. The extracted information included source and geographical context, historical period, taxonomic identity, food or consumed part, context of use, nutritional and biochemical characteristics, toxicological or antinutritional factors, traditional processing or detoxification practices, and socio-cultural or socio-political context, when reported by the source.

Because the literature included was heterogeneous, individual sources did not necessarily cover all categories. We did not infer missing information and treated it as not reported. This approach was particularly relevant for historical, ethnographic, and documentary sources, which often provide detailed contextual information but limited analytical or compositional data.

Covidence supported literature screening and data organisation. We then checked the extracted information against the original sources during manuscript preparation to verify the accuracy and contextual interpretation of the evidence. Particular attention was given to distinguishing directly reported findings from interpretations derived through cross-study synthesis.

The evidence was synthesised narratively according to the review’s thematic structure. We considered nutritional and biochemical evidence alongside toxicological, ethnobiological, historical, ecological, and socio-cultural information, while preserving distinctions between these evidence types. We did not apply formal study-quality scoring or a quantitative evidence-ranking system because the included sources differed substantially in design, purpose, historical context, and type of information provided.

A comprehensive supplementary extraction matrix was not introduced because many of the proposed fields are not applicable to historical, ethnographic, or documentary sources. Instead, relevant information is presented in the main and Supplementary Tables according to the specific evidence domains addressed by the review. This organisation allows analytical data, historical cases, traditional practices, and contextual evidence to be presented without imposing a uniform extraction structure on fundamentally different source types.

## 3. Historical Context

### 3.1. Adaptation Strategies Across Regions and Eras

The use of wild and unconventional food resources represents a long-standing human adaptation to food insecurity across diverse ecological and historical settings. Although famine foods are often associated with extreme crises, archaeological and ethnobotanical evidence suggests that reliance on supplementary wild resources has historically formed part of broader subsistence strategies [14].

Interestingly, some evolutionary and anthropological studies propose that mobile hunter-gatherer populations may have experienced fewer large-scale famines than early agricultural societies because they relied on more diversified food systems and broader ecological resource bases [40]. By contrast, agricultural dependence on a limited number of domesticated crops increased vulnerability to seasonal fluctuations, crop disease, and climatic instability.

Historical evidence further demonstrates that famine-food use has persisted well beyond pre-industrial societies. Although many of the best-known famines occurred during the nineteenth and twentieth centuries, more recent humanitarian crises show that wild plants, fungi, lichens, and other unconventional resources continue to play an important role in human survival when conventional food systems collapse. Documented examples from Bosnia and Herzegovina during the siege of Sarajevo (1992–1995) and from conflict-affected regions of Syria illustrate that famine foods remain relevant in contemporary armed conflicts and humanitarian emergencies [6,7,8].

#### 3.1.1. Historical Transitions and Regional Adaptations

During the transition from foraging to agriculture in prehistoric times, many societies continued to integrate wild foods into subsistence systems as buffers against crop failure and storage instability. Archaeobotanical evidence from East Asia indicates that early agricultural communities in Korea and Japan combined cultivated cereals with extensive use of wild nuts and gathered plant foods [41]. Similar patterns are documented in Neolithic Europe, where hazelnuts and other wild resources remained important dietary components despite the expansion of farming systems. These findings suggest that early agricultural adaptation did not involve a complete abandonment of foraging practices. Instead, cultivated and wild resources often coexisted within flexible subsistence systems designed to reduce ecological risk [42].

#### 3.1.2. Cross-Cultural and Ethnobotanical Dimensions

Adaptation to famine conditions is mediated not only by ecological availability but also by cultural knowledge systems. Ethnobotanical studies have shown that TEK strongly influences the identification, preparation, and social acceptability of wild food resources [43]. Knowledge regarding edible species, seasonal harvesting, toxicity reduction, and preservation methods is often transmitted across generations through cultural and linguistic traditions.

### 3.2. Colonialism, War, and Environmental Drivers

The transition toward famine-food consumption rarely results from environmental stress alone. Historical famines typically result from interactions among several factors, such as ecological disruption, political instability, warfare, economic inequality, and colonial intervention.

#### 3.2.1. Colonialism and Food-System Disruption

Colonial governance frequently altered indigenous agricultural systems, land use patterns, and local food economies in ways that increased vulnerability to famine. In colonial Mexico, for example, indigenous populations relied heavily on local biodiversity and traditional plant knowledge to cope with shortages associated with extractive colonial agricultural policies [44].

Similarly, scholars of the Bengal Famine have argued that British wartime policies exacerbated food insecurity by prioritising military provisioning, maintaining export-oriented systems, and inadequately responding to rural distress [45]. These dynamics transformed localised food shortages into large-scale humanitarian crises.

Comparable mechanisms also appear in northern Europe. During the Great Famine of 1696–1697, grain continued to be exported from the Swedish Baltic provinces despite severe domestic shortages, illustrating how commercial priorities and political decisions could intensify food insecurity independently of agricultural production [5]. This historical example reinforces the concept that famine often reflects failures in food access and governance rather than absolute food scarcity alone.

#### 3.2.2. War and Political Destabilisation

Armed conflict is a major driver of famine because it disrupts agricultural production, destroys infrastructure, displaces populations, and restricts food distribution networks. In some historical contexts, food deprivation has also been used deliberately as a political or military strategy [46]. Under such conditions, populations may resort to highly stigmatised or nutritionally inadequate famine foods, including unripe crops, famine grasses, and unconventional substitutes.

Recent conflicts provide well-documented examples of these adaptive responses. During the siege of Sarajevo (1992–1995), prolonged isolation forced urban populations to depend extensively on wild edible plants, mushrooms, and lichens collected from surrounding landscapes. Ethnobotanical surveys conducted after the conflict documented the consumption of numerous wild species and highlighted the critical role of local ecological knowledge in sustaining civilian populations during prolonged food shortages [6,7]. Similarly, during the Syrian conflict, communities increasingly relied on wild edible plants as conventional food supplies became inaccessible because of displacement, disrupted agriculture, and market collapse [8]. These recent case studies demonstrate that famine-food utilisation remains an important survival strategy during contemporary humanitarian crises.

#### 3.2.3. Environmental Shocks and Ecological Adaptation

Environmental disturbances—including droughts, floods, cyclones, pest outbreaks, and crop diseases—can cause famine. However, existing political and economic vulnerabilities strongly shape the effects of these shocks.

When conventional crops fail, communities often rely on drought-resistant or previously underutilised species to survive. During the 1971–1972 famine on Pemba Island, Zanzibar, for example, populations consumed toxic wild yam species such as *Dioscorea dumetorum,* which required extensive processing prior to consumption [47]. Such cases illustrate how famine-food utilisation depends not only on ecological availability but also on local knowledge systems that can reduce toxicity and maximise the use of limited nutritional resources.

### 3.3. Role of Famine Foods in Major Crises

Famine foods have historically served as emergency dietary resources during severe disruptions in food production, distribution, or access. Their consumption often reflects the collapse of conventional subsistence systems and affected populations’ inability to obtain culturally preferred staple foods. Historical evidence has demonstrated that political decisions, market disruptions, war, and unequal resource distribution frequently shape patterns of food access and survival [2,3]. Some of the major famine crises that have occurred in the world are mentioned below:

#### 3.3.1. Swedish Great Famine, 1696–1697

The Great Famine of 1696–1697 affected Sweden and neighbouring northern European regions following severe harvest failures and widespread food shortages. The crisis developed amid adverse climatic conditions that severely affected agricultural production and reduced the availability of staple grains. Historical evidence indicates that local harvest outcomes alone did not determine the consequences. Regional provisioning systems, trade conditions, and the distribution of available food resources also shaped grain availability and food access [5,13].

The Swedish case illustrates the importance of considering food access alongside agricultural production when examining historical famine. Local shortages could increase when regional supplies were insufficient or when existing provisioning networks could not compensate for production deficits. Consequently, responses to scarcity depended on both the availability of alternative food resources and the broader organisation of food distribution.

This episode also offers a historical example of how environmental stress and socio-economic conditions shape food insecurity. The Swedish experience demonstrates that famine should not be interpreted exclusively as a consequence of crop failure. Rather, the severity of food shortages may depend on how production losses interact with provisioning systems, trade, and access to available food resources [5,13].

#### 3.3.2. The Great Irish Famine (1845–1852)

The Great Irish Famine was triggered by repeated potato crop failures caused by *Phytophthora* infestans, which devastated the staple food supply of rural Irish populations. Prior to the famine, many labouring households depended heavily on potatoes and milk for daily caloric intake. When potato yields collapsed, impoverished communities increasingly relied on relief foods, imported maize, and locally available wild resources [48].

Bioarchaeological analyses of dental calculus from famine victims indicate substantial dietary dependence on imported maize distributed through relief systems [48]. Historical records also document the consumption of wild plants and emergency substitutes, including nettles, seaweed, and coarse grasses [49]. These practices reflected both severe nutritional deprivation and the collapse of traditional food entitlements among vulnerable populations.

The Irish case further demonstrates the limitations of relief efforts that introduced unfamiliar foods without adequate cultural or technical adaptation. Imported maize (“Indian meal”), widely distributed during the Great Irish Famine, was unfamiliar to many rural households and required preparation methods and cooking practices they did not understand. As a result, its acceptance was often limited and improper preparation contributed to digestive complaints among already malnourished populations, illustrating how the effectiveness of food aid depends not only on nutritional composition but also on cultural familiarity and culinary knowledge [50].

#### 3.3.3. The Bengal Famine (1943)

The Bengal Famine of 1943 resulted in widespread mortality across British India and is widely recognised as a famine intensified by wartime policy failures, inflation, market speculation, and colonial resource allocation [45]. Although environmental pressures affected regional food production, scholars have emphasised that food availability alone does not explain the scale of mortality observed during the crisis [2].

As rice prices escalated and household purchasing power declined, rural populations increasingly relied on famine foods and low-status dietary substitutes. Historical accounts describe the consumption of wild plants, famine roots, jute leaves, and improvised fillers used to extend limited grain supplies [51]. Government campaigns also encouraged “subsidiary foods” such as tapioca, yams, and groundnuts to reduce pressure on cereal reserves.

The Bengal famine illustrates how colonial governance and wartime priorities shaped inequalities in food access. Relief measures frequently failed to reach the most vulnerable populations, while market mechanisms continued to favour urban and military provisioning. In this context, famine-food consumption emerged as a survival response to both economic exclusion and institutional neglect [52].

#### 3.3.4. The Great Chinese Famine (1958–1962)

The Great Chinese Famine, associated with the Great Leap Forward, represents one of the deadliest famines in modern history, with mortality estimates commonly exceeding 30 million deaths [53]. The crisis was closely linked to collectivisation policies, unrealistic production targets, state procurement systems, and widespread disruptions to agricultural production.

As grain shortages intensified, state authorities promoted various substitute foods and emergency dietary measures intended to compensate for declining cereal availability. Historical accounts document the use of vegetable peels, wild plants, roots, tree bark, and algae-based substitutes during the crisis [54]. In some regions, rural populations were encouraged to cultivate *Chlorella* algae under improvised conditions as an alternative protein source.

The widespread reliance on unfamiliar wild resources also contributed to episodes of accidental poisoning and gastrointestinal illness. In many cases, populations lacked adequate knowledge regarding the preparation and detoxification of newly consumed plant species [54]. Therefore, TEK is crucial in mediating the safe use of famine foods during periods of extreme scarcity.

#### 3.3.5. Sarajevo and the Bosnian War (1992–1995)

Although not traditionally classified as famine, the prolonged siege of Sarajevo created conditions of severe food insecurity and restricted access to conventional food supplies that compelled civilians to rely extensively on wild edible plants, mushrooms, and lichens. Ethnobotanical investigations documented the use of numerous spontaneous plant species and highlighted the importance of Traditional Ecological Knowledge for identifying edible resources under wartime conditions [6,7]. Unlike historical agrarian famines driven primarily by crop failure, this case shows how armed conflict and prolonged disruption of food distribution can produce reliance on unconventional food resources that resembles famine-era patterns.

#### 3.3.6. The Syrian Conflict (2011–Present)

The ongoing conflict in Syria has generated prolonged food insecurity in many regions through agricultural disruption, forced displacement, and economic collapse. Ethnobotanical studies have documented the increased consumption of wild edible plants during periods when conventional foods became inaccessible or unaffordable. Species traditionally regarded as supplementary foods became essential dietary resources, demonstrating the continuing importance of local ecological knowledge in modern humanitarian emergencies [8]. These observations indicate that wild and unconventional foods may become relevant components of household food strategies during contemporary humanitarian emergencies and warrant consideration in discussions of food security and resilience.

#### 3.3.7. Ukraine: The Holodomor and Famine Foods in the 1930s

The Ukrainian famine of 1932–1933, known as the Holodomor, occurred amid collectivisation, grain procurement policies, and severe restrictions on food access. Recent ethnobotanical research documents 72 plant species and at least 114 plant parts consumed as famine foods during this period. These resources included crop residues, plants not normally eaten, and familiar plants consumed in unusual quantities or preparations. Food waste also became an important survival resource, with cereal husks, potato residues, and other discarded materials incorporated into the famine diet [21]. These practices illustrate how extreme scarcity could expand the range of plant materials incorporated into the diet, including resources normally regarded as secondary, waste, or unsuitable for routine consumption.

#### 3.3.8. Biafra: Food Scarcity and Nutritional Collapse During the Nigerian Civil War

The Biafran famine developed during the Nigerian Civil War (1967–1970), when blockade, displacement, and disruption of food supplies severely affected the population. The nutritional crisis was particularly pronounced among children, with protein deficiency and kwashiorkor becoming major concerns. Contemporary medical observations documented widespread protein malnutrition, vitamin deficiencies, and dehydration among displaced populations. Local food production remained important, particularly through crops such as maize, cassava, yams, and cocoyams, while relief programmes supplied protein-rich foods and other essential commodities [55]. This case illustrates the interaction between conflict, food-access disruption, and emergency dietary responses rather than as a detailed ethnobotanical example of famine-food use.

#### 3.3.9. Ethiopia: Wild Foods and Survival During the Famine of the 1980s

The Ethiopian famine of 1982–1985 was linked to recurrent drought, armed conflict, displacement, and disrupted rural livelihoods. As conventional food supplies declined, households progressively relied on alternative resources and carefully managed their remaining assets. Ethnographic evidence indicates that famine conditions increased the consumption of wild plants and other marginal foods, including leaves, roots, grasses, fruits, and tree-derived resources. Such foods could supplement or temporarily replace cultivated staples, despite lower sensory acceptability and, in some cases, processing or safety constraints [56,57]. These responses illustrate the importance of indigenous ecological knowledge in maintaining food access during prolonged crises. The Ethiopian experience further illustrates that famine-food use must be interpreted within the broader context of agricultural production, food access, markets, displacement, and institutional responses.

## 4. Ethnobotany and Ethnozoology

**Figure 1:**
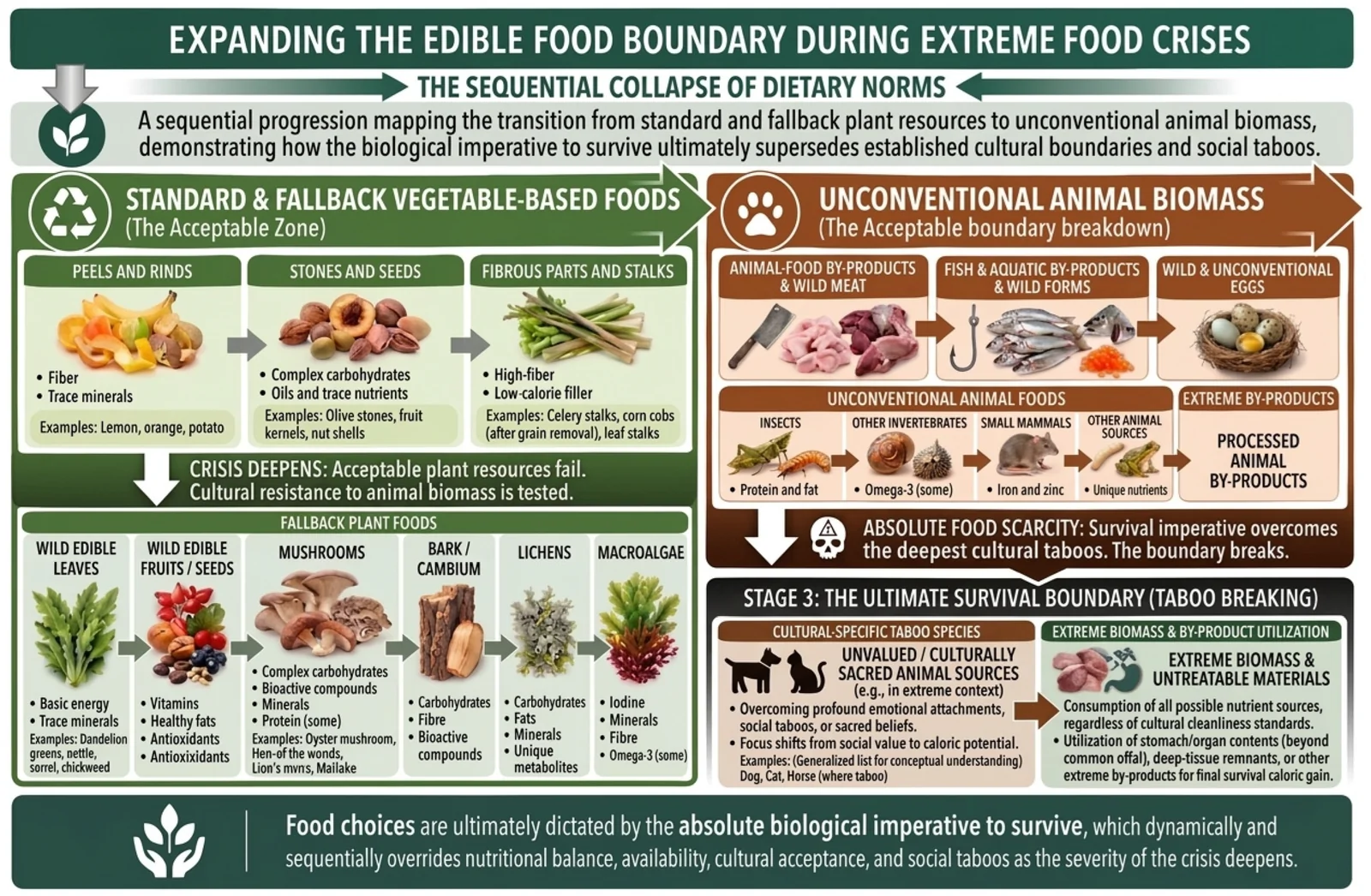
Dietary Hierarchy of Resources during Extreme Famine. Figure generated using ChatGPT (OpenAI, GPT-5.5). The authors have reviewed and edited the output and take full responsibility for the content of this publication.

A conceptual diagram presented in Figure 1 illustrates the sequential collapse of dietary norms during severe food scarcity. It maps the progression from standard and fallback plant foods to unconventional animal biomass, demonstrating how the biological imperative to survive ultimately supersedes established cultural boundaries and social taboos.

### 4.1. Wild Edible Vegetables

**Figure 2:**
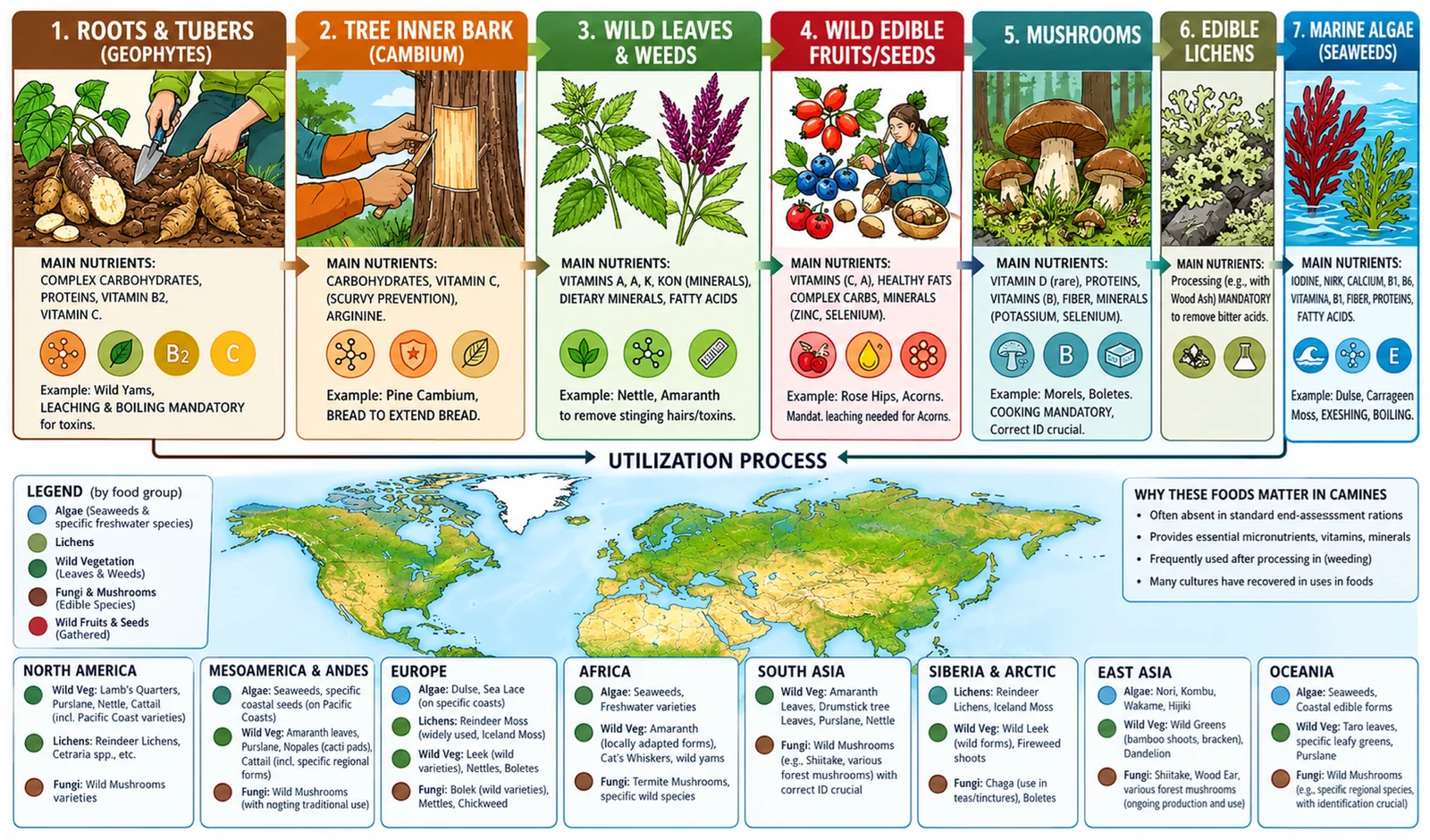
Conceptual overview of famine-food use across ecological and geographical contexts, highlighting resource categories, utilization pathways, and regional patterns.

Human populations facing famine have relied extensively on WEPs and other uncultivated biological resources to compensate for the collapse or episodic scarcity of agricultural food systems. These resources include vascular plants, fungi, algae and lichens harvested from forests, wetlands, grasslands, coastal zones, and disturbed landscapes, among others. Although they are frequently marginalised under normal socio-economic conditions, such resources become critical emergency foods during periods of food scarcity [58]. The successful use of famine flora depends heavily on ethnobotanical knowledge, particularly because many wild species contain toxic or anti-nutritional compounds that require specialised preparation prior to consumption. Traditional processing methods—including soaking, leaching, fermentation, roasting, alkaline treatment, and prolonged boiling—are therefore essential to improve digestibility and reduce toxicity. Figure 2 illustrates the major categories of wild edible vegetables commonly utilised during food shortages: roots and tubers, wild edible fruits and seeds, mushrooms, inner tree bark, wild leaves and weeds, edible lichens, and algae (seaweeds). For each type, botanical examples are presented alongside their primary nutritional contributions and the mandatory utilisation processes (such as leaching, boiling, or alkaline treatment) required to ensure safety and/or improve digestibility. The bottom half of this figure shows a global map detailing the historical and regional distribution of these plant groups and wild fungi. Regional callout boxes highlight specific ethnobotanical examples and TEK utilised across the Americas, Europe, Africa, Asia, and Oceania during times of severe scarcity.

The main food groups from wild famine vegetables are then classified and described in Table 3.

#### 4.1.1. Roots and Underground Storage Organs (USOs)

USOs, including roots, tubers, bulbs, and rhizomes, are among the most important famine-food resources because they remain physically protected beneath the soil surface during droughts, fires, and climatic disturbances [61]. These structures store starches and carbohydrates that can provide emergency caloric support when cultivated crops fail. In some cases (e.g., *Colocasia esculenta*), the leaves of the same plants can also be used [67].

Indigenous and rural communities have historically relied on wild yam species (*Dioscorea* spp.), camas bulbs (*Camassia* spp.), and other geophytes during periods of food shortage. However, many of these species contain alkaloids, tannins, oxalates, or other toxic compounds that require extensive preparation before consumption [68,69]. Detoxification methods for these USOs may involve repeated washing, prolonged soaking, roasting, or fermentation, reflecting highly specialised ecological knowledge accumulated over generations.

#### 4.1.2. Bark and Cambium

The use of inner tree bark as a food resource is documented in northern Scandinavia and other regions where seasonal or prolonged food shortages affected conventional food supplies. Among the Sami of northern Scandinavia, they traditionally harvested and used pine (*Pinus* spp.) inner bark as food. The edible material contains carbohydrates, dietary fibre, vitamin C, and minerals, which could complement other dietary sources, particularly during periods of seasonal scarcity [13]. Traditional bark harvesting involved removing selected portions of inner bark while leaving a strip of living cambium, allowing the tree to survive and reflecting established ecological and cultural knowledge [13].

The nutritional role of pine inner bark was primarily complementary rather than equivalent to that of cereal staples. Its carbohydrate content could provide energy, while fibre and micronutrients added components otherwise limited in some seasonal diets. The practice also illustrates the importance of processing and harvesting knowledge in making marginal plant resources suitable for human consumption [13].

Bark was also used as an emergency food resource in Mesoamerican contexts during severe drought. Fedick and Santiago identified the edible portion as the inner bark or phloem, a carbohydrate-transporting tissue located between the outer protective tissues and the vascular cambium. Their review of ethnographic evidence documented bark consumption as a last-resort food among Maya populations during extreme drought and identified five tree species used for this purpose. Three belonged to the Fabaceae and two to the Malvaceae [61]. The reported species included *Lonchocarpus castilloi*, *L. longistylus*, *Lepanthes guatemalensis*, *Ceiba aesculifolia*, and *Pachira aquatica* [61].

The Mesoamerican evidence further demonstrates the versatility of bark as an emergency food material. The inner bark could be eaten raw or cooked, and it could also be dried and ground into flour. Available compositional information indicates that edible bark can contain appreciable carbohydrate and protein concentrations, although nutritional data remain limited for many species [61]. Its importance during severe scarcity consequently depended not only on composition but also on the availability of suitable trees and the knowledge required for harvesting and preparation.

These examples demonstrate that bark and cambium should be considered context-dependent famine resources rather than intrinsically famine foods. Their use reflected local ecological availability, traditional knowledge, food-processing practices, and the severity of food scarcity. The Scandinavian and Mesoamerican cases also illustrate that similar emergency food strategies developed independently in distinct cultural and ecological settings [61,70].

#### 4.1.3. Wild Leaves and Herbaceous Plants

Wild leafy plants have been widely used as famine foods because they are often useful after ecological disturbance; they can provide essential micronutrients such as vitamins A and C, iron, calcium, and folate. Species such as stinging nettle (*Urtica dioica*), wild amaranth (*Amaranthus* spp.), and various ruderal weeds have historically supplemented diets during periods of scarcity [58]. Although these plants are generally less energy-dense than roots or grains, they help prevent micronutrient deficiencies often associated with prolonged famine. In many societies, however, the consumption of wild weeds and uncultivated greens carries a strong social stigma because such foods are associated with poverty and social marginalisation [71].

#### 4.1.4. Lichens and Algae

Lichens and algae occupy a unique position within famine-food systems because they are often consumed only under extreme scarcity. Certain lichen species, including *Cetraria islandica* and *Umbilicaria* spp., were historically used in northern Europe as emergency flour substitutes during crop failures. However, lichens frequently contain bitter lichen acids that require alkaline boiling or repeated washing to improve palatability and reduce gastrointestinal irritation [13].

Macroalgae provide an additional source of carbohydrates, minerals, iodine, and proteins during food crises. Some coastal communities have long harvested seaweeds such as dulse *(Palmaria palmata*) and kelps during shortages, while modern discussions of food resilience increasingly consider algae as potential emergency food resources under future climate-related disruptions [65].

### 4.2. Famine Breads and Grain Substitutes

Bread and cereal-based foods constitute dietary staples across many food cultures. Consequently, failures in cereal production can produce severe shortages of energy and staple foods. During famine conditions, populations have used a wide range of materials to extend limited supplies of cereal flour. These materials have included wild seeds, roots and rhizomes, acorns, inner bark, agricultural residues, and other locally available plant materials. Their use reflects attempts to maintain food volume and satiety when conventional grain supplies became insufficient [24,34,72]. Table 4 compares the main bread substitutes used during famine periods.

The materials incorporated into famine breads differed substantially in nutritional value, processing requirements, and potential physiological effects. Some substitutes provided appreciable carbohydrates, proteins, lipids, or micronutrients. Others contributed mainly bulk and dietary volume while offering limited nutritional value. Processing was often essential to improve palatability, digestibility, or safety. Depending on the material, preparation could involve drying, milling, leaching, washing, boiling, roasting, or fermentation [74,75,76].

The use of inner bark and cambium is also relevant to the history of famine breads. People could process pine and other tree barks into flour and incorporate it into cereal preparations when grain supplies were inadequate. Because this practice relates directly to the use of bark and cambium as famine foods, Section 4.1.2 discusses the historical background, species involved, harvesting practices, and nutritional context. Here, bark is retained only as one example of the broader range of materials that could serve as grain extenders [70].

Wild seeds and herbaceous plant materials provided another category of substitutes. Historical ethnobotanical evidence documents the use of seeds from *Amaranthus* and *Chenopodium* species as famine foods when preferred resources were unavailable. These resources could contribute carbohydrates, proteins, and minerals, although collection, cleaning, threshing, and grinding could require substantial labour [77]. Similar practices involving wild grasses and other plant materials have been reported in different regions affected by food shortages [73].

Acorns (*Quercus* spp.) represent another important category of traditional grain substitute. Acorns contain substantial amounts of starch, together with lipids, proteins, fibre, and minerals, although their composition varies among species [74,75]. Humans have used them for a long time, including in flour and bread. In Sardinia, for example, traditional acorn bread was prepared from processed acorns and incorporated locally into the diet during periods when conventional cereals were limited [76]. Acorns can contain substantial concentrations of tannins, which contribute bitterness and may reduce nutrient digestibility and bioavailability. Traditional preparation therefore commonly involved debittering procedures such as soaking, boiling, leaching, drying, or roasting before milling and food preparation [74,76].

Recent analytical research confirms that acorn flour can provide substantial starch, fibre, unsaturated fatty acids, and minerals, supporting its potential as a cereal alternative or flour ingredient [75]. At the same time, processing can modify both nutritional composition and tannin content. Leaching, for example, reduces tannins but can also alter soluble sugars, minerals, and fibre fractions [78]. Acorn should consequently not be considered a direct nutritional equivalent of cereal flour. Its dietary value depends on species, composition, processing method, and the proportion incorporated into the final food.

Under extreme famine conditions, cereal products could also contain agricultural residues or other low-nutritional-value materials. Chaff, husks, straw, wood-derived materials, and cellulose have been reported among extreme grain substitutes used when conventional ingredients were severely restricted. Historical accounts of the Siege of Leningrad describe bread formulations containing wood-derived materials and other substitutes during severe urban food shortages [68,69]. Such materials primarily increased volume or physical bulk rather than providing nutritionally equivalent substitutes for cereal grains.

Famine bread thus represents a continuum from nutritionally useful grain substitutes to emergency formulations that provide little beyond physical bulk. Their composition and nutritional significance should consequently be interpreted according to the specific material used, the processing applied, and the circumstances in which it was consumed.

### 4.3. Consumption of Unconventional Animals

Cultural customs strongly shape human food preferences regarding edibility and dietary acceptability. During periods of famine, however, these norms may shift substantially as populations seek alternative sources of protein and fat. Historical evidence demonstrates that severe scarcity frequently leads to the consumption of unconventional animal species, including insects, rodents, amphibians, companion animals, and processed animal by-products [79]. Table 5 presents a classification of the main famine foods of animal origin by animal groups.

#### 4.3.1. Entomophagy and Emergency Protein Sources

Insects are among the most nutritionally efficient famine-food resources because they provide high-quality protein, essential fatty acids, and micronutrients, including vitamins and minerals such as iron and zinc, while requiring relatively little environmental input [80]. Species such as locusts, termites, beetle larvae, and grasshoppers have historically been consumed during agricultural crises, including situations in which locust outbreaks destroyed crops while also providing emergency biomass for human consumption.

In many African, Asian, and South American Indigenous societies, entomophagy forms part of traditional dietary systems rather than constituting an exclusively famine-related practice. In Westernised societies, however, insects have often been culturally stigmatised and consumed only during severe scarcity [81].

#### 4.3.2. Rodents and Synanthropic Species

Rodents and other synanthropic animals become important famine resources because they remain available in urban and rural environments even when conventional livestock systems collapse. Historical accounts from the Great Chinese Famine and wartime Europe describe consuming rats and mice as an emergency protein source [79]. Although nutritionally valuable, wild rodents also pose substantial epidemiological risks because of their association with zoonotic diseases and parasites. Thorough cooking and preparation, therefore, become essential for reducing infection risks during famine conditions [82].

#### 4.3.3. Companion Animals and Captive Fauna

Consuming companion animals and zoo fauna represents one of the most socially and psychologically significant thresholds in famine adaptation. During extreme sieges and urban famines, populations have historically consumed dogs, cats, horses, and captive zoo animals after conventional food sources were exhausted [79]. Historical examples such as the Sieges of Paris or Leningrad illustrate how prolonged starvation can override deeply embedded cultural prohibitions regarding animal consumption [68,69]. These cases demonstrate the extent to which famine restructures social norms surrounding edibility and survival.

#### 4.3.4. Leather and Animal By-Products

In the most severe famine conditions, populations may consume processed animal by-products such as leather belts, shoes, harnesses, and hides. Extensive boiling can partially hydrolyse collagen into gelatin, which has a minimal caloric and nutritional value but temporary satiety [53]. Consuming these food products illustrates the final stages of food-system exhaustion, in which populations rely on previously inedible manufactured materials for survival.

### 4.4. Indigenous Knowledge Systems and Survival Practices

WEPs form a critical component of indigenous survival systems because they are frequently adapted to local climatic variability and environmental stress. In drought-prone regions such as Ethiopia, communities continue to use drought-resistant WEPs to supplement diets during seasonal and chronic food shortages [83]. Knowledge regarding edible species, seasonal availability, toxicity reduction, and preparation techniques is often highly specialised and transmitted across generations through oral traditions and cultural practices.

The continued use of famine foods depends heavily on cultural memory and intergenerational transmission of TEK. Communities that maintain historical knowledge of edible landscapes are often better equipped to respond to environmental and economic disruptions. So, some studies in Mesoamerica and other regions have shown that biocultural relationships with local ecosystems contribute significantly to resilience during food crises [44]. In this context, famine-food knowledge functions not only as a survival mechanism but also as an important component of cultural identity and continuity.

During famine conditions, TEK often determines whether wild biological resources can be transformed into viable food sources. Despite its adaptive importance, TEK is increasingly threatened by urbanisation, industrial agriculture, land-use change, and the social stigmatisation of foraging practices [84]. Colonial policies and modernisation processes have further disrupted Indigenous food systems and weakened the transmission of ecological knowledge.

At the same time, growing interest in food sovereignty, biodiversity conservation, and sustainable food systems has renewed attention to Indigenous knowledge and traditional wild-food practices. Revitalisation efforts increasingly recognise TEK as an important resource for strengthening food-system resilience under conditions of climate change and ecological uncertainty [85].

## 5. Nutritional and Toxicological Aspects

### 5.1. Nutritional Value of Famine Foods

The presence of a toxic or antinutritional constituent is considered a potential hazard rather than evidence of toxicological risk by itself. Risk depends on concentration, dose, exposure, processing, frequency of consumption, and consumer susceptibility. The literature reviewed here distinguishes, where information permits, intrinsic toxicity, contamination, antinutritional factors, allergenicity, and hazards associated with animal-derived foods.

A persistent concern within famine-food scholarship is the romanticisation of “wild nutrition”. Famine foods occupy a nutritional paradox within human survival systems. On one hand, many wild plants, insects, algae, and unconventional animal species offer remarkable nutritional value, with high concentrations of micronutrients, essential fatty acids, and bioactive compounds. On the other hand, these resources are often seasonally scarce, labour-intensive to procure, difficult to digest, or physiologically unsafe when consumed without adequate and specialised preparation. Consequently, the nutritional value of famine foods must be evaluated not only by their biochemical composition, but also by their bioavailability, toxicity, energetic harvesting and preparation costs, and long-term sustainability during prolonged crises.

Numerous ethnobotanical studies have emphasised the superior nutritional and phytochemical richness of wild taxa compared with commercial crops, yet many analyses overlook the practical realities of famine physiology. Starving populations rarely consume these foods under ideal conditions; instead, they consume them while dehydrated, infected, micronutrient-deficient, and under severe caloric stress. Thus, the theoretical nutritional superiority of many famine foods does not necessarily translate into improved nutritional status during famine conditions [14,25].

#### 5.1.1. Wild Botanical Sources: Nutritional Density and Ecological Resilience

Supplementary Table S1 [63,66,70,86,87,88,89,90,91,92,93,94,95,96,97,98,99,100,101,102,103,104,105,106,107,108,109,110,111,112,113,114,115,116] shows the caloric contribution (kcal per 100 g) and nutritional composition, expressed in g or mg per 100 g of non-animal-origin famine foods. This composition varies with the organ or part of the vegetable considered. In general, moisture was the major component, representing >80% in most of the famine foods described. Obviously, the lowest mean moisture contents were observed when concentrations were expressed on a dry-weight basis, for example, for lichens, inner bark, or seeds. It is remarkable that some wild fruits, such as strawberry tree fruit and rosehip, presented relatively low contents, around 50%. Caloric and moisture contents showed inverse behaviour. Many famine plants are nutritionally impressive, but only when they are evaluated on a dry-weight basis. During actual famine conditions, however, foods are consumed fresh, diluted, or incompletely processed, which significantly reduces nutritional and caloric value.

Carbohydrates were the most abundant macronutrient, ranging between 1 and 10%. Lichens, bulbs of Sego lily, and the previously mentioned wild fruits showed the highest mean carbohydrate contents, due to their lower moisture content. In many famine foods, their consumption contributes considerably to the fibre intake. The mean protein content was relatively low, ranging from 0.5 to 3%, except for seeds, which showed higher values. Most of the vegetable famine foods also showed very low lipid concentrations (<0.5%), but their fatty acid profiles are nutritionally interesting. Polyunsaturated fatty acids were very abundant, and the n-6 LA: n-3 ALA ratio was between 0.5 and 10, which has been recognised as beneficial for human health [117].

WEPs frequently exhibit greater concentrations of micronutrients (vitamins and minerals), phytochemicals, antioxidants, and secondary metabolites than highly domesticated crops. This is because they have not undergone selective breeding, primarily for yield, sweetness, or storage stability [58,118]. Many famine plants therefore function as critical micronutrient buffers during crises, especially in preventing deficiency diseases such as scurvy, anaemia, and pellagra.

Water-soluble vitamins are of greater nutritional importance than fat-soluble vitamins, with vitamin C being particularly notable. Many famine foods of vegetal origin contribute significantly to the intake of this antioxidant vitamin [101]. The contribution of B vitamins is also notable, particularly folates. Considerable mineral concentrations were observed, with ash content ranging from 1 to 6%; the main minerals present were potassium, phosphorus, calcium, magnesium, iron, and zinc, as well as iodine and selenium in marine macroalgae [66]. Notably, the wide variety and high concentrations of bioactive compounds, such as peptides, taurine, anserine, creatine, chitin, and coenzyme Q10, vary by animal species [119,120].

As shown in Supplementary Table S1, species such as *Sonchus oleraceus* and *Chenopodium album* contain substantially higher concentrations of vitamin C, iron, and omega-3 fatty acids than conventional leafy vegetables. In addition, *Chenopodium album*, for example, has been reported to contain α-linolenic acid and several micronutrients and phytochemicals, alongside elevated carotenoid and tocopherol contents [95,96]. Likewise, purslane (*Portulaca oleracea*) is widely recognised as one of the richest terrestrial plant sources of omega-3 fatty acids, despite historically being stigmatised as a weed [121].

Wild fruits similarly provide an exceptional phytochemical density. Rosehip (*Rosa canina*) contains extraordinarily high concentrations of vitamin C and carotenoids compared with cultivated fruits such as plums or apples [103,104]. However, wild fruits are highly seasonal, difficult to preserve without technological infrastructure, and often geographically restricted. Consequently, despite their impressive biochemical profiles, their capacity to sustain populations during prolonged famines is limited.

Roots, tubers, and bark serve primarily as emergency caloric resources rather than as balanced nutritional foods. The inner bark of *Pinus sylvestris* contains substantial complex carbohydrates and fibre, allowing it to extend flour supplies during cereal shortages [70]. Similarly, wild geophytes such as sego lily bulbs provide concentrated starch reserves capable of sustaining short-term caloric survival [111]. Nevertheless, these foods also reveal the physiological compromises inherent in famine adaptation. Pine bark flour, for instance, is poorly digestible because of its lignin content and may cause severe gastrointestinal distress, constipation, and intestinal irritation when it is chronically consumed [13].

Marine algae and lichens represent additional examples of ecologically resilient famine foods. Seaweeds such as *Palmaria palmata* supply iodine, potassium, magnesium, and marine omega-3 fatty acids, making them valuable coastal survival resources [66]. Yet excessive reliance on seaweeds may simultaneously induce iodine toxicity or heavy-metal exposure, depending on species and environmental contamination [66]. Likewise, lichens such as *Cetraria islandica* historically prevented starvation in northern Europe, but their bitterness, poor digestibility, and high acidity required extensive alkaline processing before consumption [13].

#### 5.1.2. Zoological Sources: Protein Density and Survival Efficiency

Nutritional composition, expressed in g or mg per 100 g, and caloric content (kcal per 100 g) of some unconventional animals sometimes used as famine foods are presented in Supplementary Table S2 [22,28,119,120,122,123,124,125,126,127,128,129,130,131,132,133,134,135]. Substantial differences can be observed when these famine foods were nutritionally compared with famine foods of vegetable origin. Famine foods of animal origin generally provide more bioavailable proteins, lipids, and micronutrients. Insects, rodents, molluscs, amphibians, and small fish constitute highly efficient survival foods because they offer dense nutrient packages relative to their biomass and ecological footprint [22]. The chemical composition varies depending on the animal family and/or genus considered. Moisture content in animal-based famine foods was lower than in vegetables, ranging from 50 to 70%, except for amphibians, fish, and shellfish. With respect to the macronutrients, proteins were the major fraction, which presents a relatively high biological value. In most of these famine foods, the observed content ranged from 18 to 22%. Leather had the highest protein concentration and the lowest water content. The lipid content was relatively high and highly variable across many animal species, and the proportion of fatty acids was also variable, consistent with the high caloric content of these foods of animal origin. In contrast, the carbohydrate fraction is very low in foods of animal origin.

Fat-soluble vitamins, such as tocopherols and vitamin D (in fish), were present in these famine foods; complex B vitamins, including B12, were also present, while vitamin C was absent. Considerable mineral concentrations were observed, with an ash content ranging from 1 to 6%; the main minerals were electrolytes (sodium and potassium), phosphorus, calcium, magnesium, iron, and zinc, as well as selenium and iodine in the seawater animal species. The wide variety and high concentration of bioactive compounds, such as peptides, taurine, anserine, creatine, chitin, and coenzyme Q10, also vary by animal species.

Among arthropods, termites and palm weevil larvae are particularly notable for their high lipid concentrations and caloric density. Palm weevil larvae may exceed 500 kcal per 100 g, making them among the most energy-rich famine foods documented ethnographically [123]. Similarly, locusts and grasshoppers provide considerable amounts of protein, iron, zinc, and unsaturated fatty acids [122]. During locust infestations, these insects paradoxically function simultaneously as agricultural destroyers and emergency nutritional resources.

Nevertheless, nutritional enthusiasm surrounding entomophagy must be critically contextualised. The digestibility of insect protein is substantially reduced by the presence of chitin, while harvesting efficiency varies dramatically by species, season, and ecology [123]. Moreover, many studies promoting insects as sustainable proteins were conducted under controlled laboratory conditions and do not adequately reflect the sanitary conditions of famine environments, where contamination, pesticide accumulation, and microbial spoilage are commonly present [136].

Rodents and amphibians provide complete proteins with relatively favourable amino acid profiles. Frogs additionally contain biologically active antimicrobial peptides such as magainins and dermaseptins [128]. Small fish and molluscs are particularly valuable because they provide omega-3 fatty acids, taurine, calcium, vitamin D, and trace minerals, which are often absent from terrestrial famine diets [119].

However, the nutritional benefits of unconventional animal foods are inseparable from their epidemiological risks. Rodents are major vectors of zoonotic diseases, while molluscs readily accumulate marine toxins, contaminants, and pathogenic microorganisms. Thus, their nutritional utility depends heavily on adequate cooking, ecological knowledge, and sanitary handling, which are often absent during famine crises [82].

The consumption of leather and processed animal hides illustrates the extreme endpoint of nutritional desperation. Although boiled leather contains measurable protein derived from collagen, its amino acid profile is severely incomplete and biologically inefficient [132]. In practical terms, leather consumption represents not a nutritional adaptation but a metabolic collapse. Its historical use demonstrates that famine foods often function psychologically by producing temporary satiety rather than physiologically by supporting meaningful recovery.

### 5.2. Anti-Nutritional and Toxic Compounds and Toxins Factors

Many famine resources remain outside conventional diets precisely because they contain toxic compounds, anti-nutritional agents, allergens, or infectious hazards that render them physiologically dangerous under normal conditions. During famine, however, starvation forces populations to renegotiate acceptable risk thresholds, transforming biologically hazardous materials into emergency foods.

While famine foods provide life-saving calories and associated macro- and micronutrients, their utilisation presents a severe physiological paradox: starving populations must expend critical, rapidly depleting energy reserves to neutralise the potent defensive compounds inherent in wild species. For example, a plant rich in micronutrients may still be maladaptive if collecting and detoxifying it requires more energy than it provides. Likewise, some high-protein famine foods increase renal stress or dehydration under conditions of inadequate water intake. The toxicological dimension of famine foods is central to understanding their historical role in human survival. Thus, it is not something merely biochemical; it is necessary to approach it from a political, ecological, and anthropological perspective [137].

This survival paradox reveals an important principle: famine foods are often not truly “safe foods,” but rather foods that become temporarily tolerable through labour-intensive cultural processing and accumulated TEK [24]. Without this knowledge, famine foods may accelerate mortality rather than prevent it. Supplementary Table S3 shows selected toxic and anti-nutritional compounds in famine foods, their effects on health, and mitigation treatments [36,80,121,123,138,139,140,141,142,143,144,145,146,147,148,149,150,151,152,153,154].

#### 5.2.1. Inherent Plant Toxicity, Chemical Defence Systems and Epidemiological Hazards

Many wild famine plants evolved potent chemical defence systems to deter herbivory. These compounds are secondary metabolites, including alkaloids, cyanogenic glycosides, oxalates, tannins, terpenoids, and neurotoxins, that pose immediate and severe risks to human health. Under normal conditions, people avoid these plants or consume them only after careful preparation. In addition, famine and starvation often compromise or eliminate essential detoxification processes. Bitter cassava (*Manihot esculenta*) represents one of the most significant examples; it contains cyanogenic glycosides, specifically linamarin and lotaustralin, which can trigger acute cyanide poisoning [36,138]. These glycosides can release lethal hydrogen cyanide when improperly processed. Chronic consumption of inadequately detoxified cassava is strongly associated with konzo, a permanent upper motor neuron disease linked to cyanide exposure and sulfur amino acid deficiency [36,138]. Importantly, outbreaks of cassava toxicity disproportionately affect populations already weakened by protein malnutrition, illustrating how famine intensifies vulnerability to toxins.

Bracken fern (*Pteridium aquilinum*) presents an especially important example because it demonstrates how famine adaptation may generate long-term carcinogenic effects. Although historically consumed across Europe and Asia, bracken contains ptaquiloside, a known carcinogen that has been associated with gastrointestinal and bladder cancers [141]. These examples complicate simplistic narratives of famine resilience by showing that survival foods can produce delayed chronic disease burdens. Grass pea (*Lathyrus sativus*) becomes heavily relied upon during drought because of its remarkable climatic resilience. Yet prolonged dependence on this crop, or if it is improperly processed, can induce neurolathyrism—an irreversible spastic paralysis—through exposure to β-ODAP neurotoxins [140]. Furthermore, cycad seeds contain potent neurotoxins cycasin and β-N-methylamino-L-alanine (BMAA), consumption of which is linked to severe neurodegenerative diseases [147]. This creates a tragic agricultural paradox: some of the most famine-resistant crops are simultaneously among the most neurologically dangerous. Likewise, wild yams (*Dioscorea* spp.) contain toxic alkaloids, including dioscorine [143]. Other toxic compounds present in some WEPs, such as saponins and nitrates, can also produce adverse health effects. Excessive nitrate accumulation in certain ruderal weeds may, under specific environmental conditions, increase the risk of nitrate-induced methemoglobinemia following in vivo conversion to nitrite, particularly in vulnerable individuals. In addition, nitrate accumulation is strongly influenced by environmental factors such as nitrogen availability, drought stress, and reduced light intensity [155,156].

Many famine foods contain high amounts of anti-nutritional factors that impede human digestion and exacerbate malnutrition. Wild greens commonly used during scarcity also contain anti-nutritional compounds that impair nutrient absorption. So, *Chenopodium* species and purslane (*Portulaca oleracea*) are rich in oxalates, which actively bind to dietary calcium and induce malabsorption of calcium and other minerals [121,145]. Acorns (*Quercus* spp.) contain high levels of hydrolysable tannins, which drastically reduce protein digestibility and cause mineral chelation [139].

#### 5.2.2. Toxicological Risks in Animal Farming Foods

Insects, despite their high nutritional efficiency, are not toxicologically neutral. Chitin may reduce protein digestibility and provoke allergic responses, especially among individuals sensitised to crustaceans [148]. Locusts and grasshoppers also pose risks of pesticide bioaccumulation in modern agricultural landscapes. Thus, promoting entomophagy today must distinguish between controlled food systems and emergency famine harvesting.

Animal-sourced famine foods pose a different spectrum of hazards, dominated by infectious diseases, parasites, environmental contamination, and allergenicity. Unlike cultivated livestock systems, wild famine fauna is typically consumed without veterinary oversight, sanitary infrastructure, or pathogen control [82]. Unconventional animal foods also present digestive challenges; the high chitin content in termites and beetle larvae reduces overall human digestibility, while locusts contain high levels of purines [80,123,148].

Shellfish, particularly bivalve molluscs, may accumulate marine biotoxins such as paralytic shellfish toxins (PSTs) and domoic acid during harmful algal blooms, while also bioaccumulating heavy metals from contaminated aquatic environments [153]. Likewise, improperly preserved fish rapidly accumulate histamine through bacterial decarboxylation of histidine, increasing the risk of scombroid poisoning [154]. Because populations experiencing famine often lack the opportunity to selectively harvest or safely preserve aquatic resources, the risk of foodborne intoxication and poisoning may increase substantially during periods of crisis.

Rodents constitute a particularly important case because they combine high nutritional value with substantial epidemiological risk. Wild rats and mice act as reservoirs for leptospirosis, hantavirus, salmonellosis, and numerous parasitic infections [151]. Historical famines repeatedly document rodent consumption during urban sieges and agricultural collapse, yet famine historiography has largely left the public health consequences of this practice underexamined.

Animal sources introduce significant toxicological and infectious hazards. Foraging for unconventional fauna also carries massive epidemiological risks; wild rodents and reptiles act as primary vectors for severe zoonotic pathogens, including *Leptospira*, hantavirus, and *Salmonella* [151], while freshwater snails transmit trematode parasites like *Fasciola* [149].

Amphibians and reptiles can introduce several hazardous substances through their skin, including alkaloids, parasites, and bacterial contamination. Some frog species produce potent neurotoxins, while reptiles are major carriers of *Salmonella* spp. [150]. These risks are amplified when famine conditions limit the availability of fuel for thorough cooking.

#### 5.2.3. Mandatory Mitigation Strategies

To make these potentially hazardous resources biologically viable, populations must employ rigorous, labour-intensive mitigation strategies. Bitter cassava requires extensive fermentation, grating, and soaking to volatilize hydrogen cyanide [36]. Acorns must undergo prolonged water leaching followed by roasting to remove tannins [139]. Toxins in bracken fern and Iceland moss can be partially mitigated through repeated boiling in alkali solutions, discarding the water [141,142]. For animal sources, thorough, prolonged cooking, complete skin removal, and strict hygiene and sanitation are critical to neutralise parasites, bacteria, and thermolabile toxins.

#### 5.2.4. Critical Limitations in the Nutritional Interpretation of Famine Foods

A major weakness in famine-food literature is the tendency to evaluate foods in isolation rather than within integrated famine ecologies. Nutritional composition alone does not determine survival value. Multiple variables critically shape the usefulness of famine foods, including the energetic cost of harvesting, seasonal availability, digestibility, toxin burden, preparation time, fuel and water requirements, cultural acceptance, and disease exposure.

Another major concern is the frequent comparison between wild and cultivated foods, which uses modern analytical standards detached from historical realities. While many famine foods outperform commercial vegetables nutritionally, people rarely consumed them voluntarily. Their ingestion usually reflected institutional collapse, coercion, and desperation rather than dietary preference. Therefore, famine foods should not be idealised as inherently superior alternatives to cultivated agriculture. Instead, they should be understood as biologically and culturally complex emergency resources that simultaneously embody resilience, adaptation, toxicity, labour burden, and social trauma [21].

#### 5.2.5. The Energetics for Detoxification of Famine Foods

The economic logic of famine foraging contains an important contradiction. Many famine foods require prolonged soaking, boiling, fermentation, grinding, or alkaline treatment before they become safe to eat. These processes demand water, fuel, time, and physical energy—resources often critically scarce during famine conditions. Moreover, this extraordinary labour investment occurs precisely when starvation is physiologically weakening affected populations. For example, safely preparing cassava may require multiple days of soaking and fermentation. Acorn detoxification similarly requires repeated water leaching to remove tannins. Lichens often require alkaline boiling with wood ash to neutralise bitter acids [142]. In some cases, the caloric expenditure required to obtain and process these resources approaches—or even exceeds—the calories ultimately gained from their consumption. These procedures impose major metabolic costs on populations already experiencing caloric collapse. Thus, famine foods frequently represent not efficient survival solutions, but desperate, diminishing-return coping strategies imposed by structural deprivation.

## 6. Socio-Cultural Dimensions

Famines are not solely crises of caloric and nutritional insufficiency; they are profound social and cultural ruptures that reshape identities, relationships, moral systems, and collective memory. The consumption of famine foods, therefore, cannot be interpreted merely as a nutritional adaptation. Rather, these foods operate simultaneously as biological resources, social symbols, and political indicators of exclusion and vulnerability [2,21,50].

Importantly, famine-food scholarship has historically been dominated by nutritional and economic analyses, often neglecting the emotional, symbolic, and cultural dimensions of eating under duress. Yet anthropological evidence demonstrates that famine transforms not only what people eat, but also what food means socially and morally [34,157]. In this sense, famine foods are embedded within broader systems of identity, inequality, memory and power. Their use reveals how communities negotiate dignity, memory, stigma, and survival under conditions of extreme structural failure.

### 6.1. Stigma and Memory Associated with Famine Foods

The consumption of famine foods is deeply associated with humiliation, bodily suffering, and social degradation. Because these resources are generally consumed only during periods of extreme deprivation, they frequently become enduring cultural markers of poverty and social collapse. Survivors often retain vivid sensory memories of famine foods—including bitterness, foul odours, difficult textures, or gastrointestinal distress—which become permanently linked to trauma and fear [158].

Consequently, many famine foods acquire a powerful social stigma long after the crisis ends. Foods once consumed for survival are retrospectively categorised as “poverty foods,” “foods of shame,” or symbols of backwardness [159]. This stigmatisation extends beyond the foods themselves to encompass the social identities associated with them. Rural populations, indigenous groups, and historically marginalised communities may therefore experience cultural devaluation because of their historical association with foraging or substitute foods. The social rejection of famine foods frequently accelerates dietary westernisation and nutritional transition following famine recovery. Survivors often abandon traditional ecological resources in favour of industrialised ones because modern foods symbolise social advancement, urbanisation, and escape from hunger. This phenomenon demonstrates that food choices are shaped not only by nutrition, but also by aspiration, memory, and class identity [27].

However, the relationship between memory and famine foods is highly complex and politically mediated. In many authoritarian or colonial contexts, famine experiences were actively silenced or erased from official narratives. During the Spanish “*años del hambre”* following the Civil War, for example, public discussion of starvation and substitute foods was heavily suppressed, forcing famine experiences into private memory rather than collective acknowledgement [160]. Similar patterns occurred during the Great Chinese Famine, where state narratives minimised or denied starvation despite widespread consumption of wild plants, bark, and ersatz foods [53].

This politicisation of memory is significant because it shapes how famine foods are remembered across generations. In many societies, older generations retain embodied memories of famine, while younger generations inherit only fragmented narratives or silence. As a result, mainstream food histories often omit the historical role of famine foods despite their profound demographic importance [21].

At the same time, stigma surrounding famine foods is not universal or static. Some formerly stigmatised foods have undergone cultural revalorization in modern contexts. Seaweeds, wild greens, fermented foods, and edible insects—once associated with scarcity—are increasingly marketed as sustainable, artisanal, or nutritionally superior foods within affluent societies. This transition exposes an important sociological contradiction: foods historically consumed out of desperation may later become commodified as luxury or health products once detached from their famine associations [14,19,27].

### 6.2. Role in Identity, Resilience, and Oral History

Famine foods also function as repositories of cultural memory and resilience. In many indigenous and rural societies, knowledge regarding edible wild species, detoxification methods, seasonal harvesting, and ecological indicators constitutes an essential component of TEK [24].

This knowledge is rarely transmitted through formal written systems. Instead, communities preserve it through oral histories, ritual practices, storytelling, apprenticeship, and intergenerational observation. Such transmission enables communities to maintain ecological flexibility during periods of environmental instability and agricultural failure [44].

Importantly, famine-food knowledge often survives precisely because communities repeatedly encounter ecological uncertainty. In drought-prone, mountainous, or politically marginalised regions, familiarity with fallback foods becomes a long-term adaptive strategy rather than merely an emergency response. Thus, famine foods should not always be interpreted exclusively as signs of collapse; in some contexts, they represent accumulated resilience within highly variable ecosystems [14,161].

However, this knowledge is increasingly threatened. Urbanisation, land dispossession, formal schooling systems, and industrialised agriculture have disrupted intergenerational ecological learning across many societies [84]. Younger generations may therefore lose the practical skills required to identify, harvest, and safely prepare famine foods.

At the same time, contemporary movements toward food sovereignty and cultural revitalisation have begun reclaiming some traditional famine foods as markers of identity and resistance. Indigenous communities, environmental activists, and local food movements increasingly reinterpret wild foods as symbols of ecological sustainability, cultural continuity, and resistance to industrialised agriculture [159].

Oral histories play a particularly important role in preserving this important heritage. Narratives surrounding famine foods frequently transmit not only survival strategies, but also moral lessons concerning scarcity, reciprocity, social solidarity, and institutional betrayal. In this way, famine memory becomes embedded within family histories and collective identity [21]. Moreover, oral traditions often preserve information omitted from official archives. Government records may document grain prices or mortality rates, but survivors’ narratives reveal the lived experiences of scavenging, shame, food substitution, and social fragmentation.

### 6.3. Gender and Social Inequalities in Access to Food

Famines expose and intensify pre-existing social inequalities. Access to food during crises is rarely distributed equally; rather, it is shaped by class, gender, ethnicity, age, political status, and social power. Consequently, famine foods become embedded within unequal systems of labour, entitlement, and bodily sacrifice [50,162].

Gender inequalities are especially significant. Across numerous historical and ethnographic contexts, women bear disproportionate responsibility for food acquisition, preparation, and household survival during famine conditions [163]. This includes labour-intensive tasks such as foraging, carrying water, gathering fuelwood, detoxifying plants, and preparing substitute foods. These activities substantially increase women’s physical workload precisely when caloric intake is declining. Intra-household food distribution is similarly unequal. In many patriarchal systems, adult males receive priority access to limited food resources because of cultural norms linking male labour with household survival [163]. Women, children, and the elderly may therefore experience more severe nutritional deprivation despite participating heavily in food procurement and preparation. These disparities become especially acute during prolonged crises. Chronic undernutrition among women contributes to maternal mortality, impaired lactation, reproductive complications, and intergenerational malnutrition. Thus, famine produces long-term demographic and developmental consequences that extend beyond immediate starvation mortality [164].

Class and political inequalities further shape access to famine foods. Wealthier households often retain greater access to land, livestock, storage infrastructure, and social networks, enabling them to delay or avoid reliance on stigmatised substitute foods. Poorer households, by contrast, are forced earlier into high-risk survival strategies such as scavenging, rodent consumption, or migration [165].

Importantly, famine foods themselves may become stratified according to status. Some substitute foods are accessible only to those with ecological knowledge, mobility, or rights of access to forests, rivers, or coastlines. Colonial land policies and environmental enclosure have historically restricted such access for indigenous and peasant communities, thereby undermining traditional survival systems [24].

Social fragmentation during famine further intensifies inequality. Anthropological studies have repeatedly documented the collapse of reciprocity and communal food sharing under extreme scarcity [157]. As hunger intensifies, households prioritise immediate survival over collective obligations, leading to social atomization, theft, abandonment, and exclusion of vulnerable individuals.

## 7. Environmental and Ecological Perspectives

The relationship between famine foods and the environment reveals a central paradox of human survival: the same ecosystems that sustain populations during catastrophic food shortages are frequently degraded by the very extraction pressures that crises generate [14]. Famine foods can be considered ecological resources embedded within broader systems of biodiversity, climate variability, land use change, and environmental governance. Their utilisation demonstrates both the adaptive capacity of human societies and the structural fragility of food systems dependent on narrow agricultural monocultures [166]. In addition, the erosion of TEK has important implications for future climate resilience, as many adaptive survival systems depend on localised ecological expertise that is unavailable through centralised food systems.

Contemporary scholarship increasingly critiques state-centred food security paradigms for overemphasising agricultural productivity while underestimating the buffering role of unmanaged ecosystems and wild biological diversity. In practice, many rural and marginalised populations survive food shortages not through formal relief systems alone, but through access to forests, wetlands, coastal environments, and uncultivated landscapes that provide fallback foods during periods of crop failure [14,167]. Yet this dependence also highlights a profound ecological contradiction, as the availability of these emergency resources declines as climate change, biodiversity loss, and land degradation intensify.

### 7.1. Biodiversity as a Buffer Against Famine

Biodiversity functions as a critical biological insurance system during episodes of food-system collapse. Diverse ecosystems provide access to uncultivated edible plants and unconventional animals, which can temporarily compensate for failures in domesticated agriculture. Ethnobiological studies have demonstrated that many forager and agrarian societies have routinely utilised dozens—sometimes more than one hundred—wild species within a single ecological region, particularly during seasonal shortages or crop failures [14]. These species collectively expand the dietary base beyond the narrow dependence imposed by industrial monocultures.

This ecological diversity matters because wild taxa exhibit highly variable phenologies, reproductive strategies, drought tolerances, and ecological niches. Unlike standardised commercial crops, which are often genetically uniform and vulnerable to synchronised failure, wild food species respond differently to environmental stressors. Consequently, environmental shocks that devastate one resource may leave others available, thereby reducing the probability of complete subsistence collapse. This ecological heterogeneity provides what resilience theorists describe as “functional redundancy,” whereby multiple species can fulfil similar nutritional roles under changing environmental conditions [168].

WEPs are particularly significant during the annual “hunger season,” when stored grains are depleted, and new harvests remain unavailable. During these periods, communities frequently intensify the collection of alternative and uncultivated plant foods such as roots, leafy greens, fruits, seeds, and aquatic resources to bridge caloric deficits [167]. Therefore, during food crises, biodiversity can contribute not only to the caloric intake but also to the nutritional quality of the diet.

However, romanticising biodiversity as an automatic safeguard against famine warrants critical scrutiny. Access to biodiverse environments is profoundly unequal and mediated by land ownership, state regulation, conflict, and environmental degradation. Populations displaced by war, colonial land dispossession, conservation exclusion policies, or urbanisation may be physically separated from the ecosystems upon which famine resilience depends. Moreover, biodiversity itself cannot compensate indefinitely for systemic political failures such as unequal food distribution, armed conflict, or economic exclusion. Ecological abundance may mitigate famine risk, but it does not eliminate the structural causes of starvation [169].

### 7.2. Climate Variability and Ecosystem Dependence

Climate variability increasingly intensifies human dependence on wild ecosystems as conventional agricultural systems become less predictable. Prolonged droughts, altered precipitation regimes, extreme heat, shifting pest distributions, and erratic seasonal cycles destabilise crop yields in vulnerable regions. Under these conditions, populations frequently turn toward uncultivated biological resources that exhibit greater ecological resilience than highly domesticated crops [118,170].

Many famine foods derive from species naturally adapted to marginal or unstable environments. For example, wild roots survive seasonal droughts through subterranean storage structures; drought-resistant shrubs persist where cereals fail; marine algae and intertidal resources remain available even during terrestrial agricultural collapse. Because these species evolved under fluctuating environmental conditions without reliance on irrigation, fertilisers, or mechanised cultivation, they often show greater tolerance to ecological stress than industrial crop varieties optimised primarily for yield maximisation [14,168].

This adaptive resilience creates a profound dependence on surrounding ecosystems during climatic crises. Studies indicate that communities located near forests, wetlands, and protected transitional ecosystems often maintain greater dietary diversity and improved nutritional outcomes during periods of food insecurity. Forest margins, in particular, provide edible fruits, leaves, fungi, bushmeat, medicinal plants, and fuelwood necessary for processing famine foods safely [167].

Nevertheless, climate change simultaneously erodes the ecological foundations of these coping systems. Rising temperatures, altered rainfall patterns, salinisation, desertification, the expansion of invasive species, and increased wildfire frequency increasingly threaten the habitats that sustain emergency food resources. Many wild species have narrow ecological thresholds and may experience declining reproductive success or geographic displacement in response to rapid climatic shifts. Consequently, vulnerable populations face what can be described as a “double ecological exposure”: climate change destabilises agricultural production while concurrently degrading the fallback ecosystems required for survival [171].

On the other hand, ecosystem dependence itself may intensify social inequalities. Communities with secure land tenure, mobility, and ecological knowledge are better positioned to exploit wild resources during crises, whereas displaced populations, refugees, and the urban poor often lack both TEK and access to it. Thus, climate-driven ecosystem dependence does not affect all populations equally; rather, it amplifies existing disparities in environmental access, political marginalisation, and adaptive capacity [169].

### 7.3. Overharvesting Risks During Crises

Although natural ecosystems serve as emergency food reserves during famines, desperation-driven extraction often causes severe ecological degradation. Under conditions of acute starvation, long-term sustainability becomes subordinate to immediate biological survival. As a result, harvesting practices that may be sustainable under normal conditions can rapidly become destructive when intensified across entire populations [14]. This shift is especially evident in the transition from harvesting renewable plant tissues to extracting non-renewable survival organs. Under ordinary circumstances, foragers may primarily collect leaves, fruits, or seeds that allow plants to continue reproducing. During famines, however, populations increasingly target calorie-dense roots, tubers, bulbs, and tree cambium because these structures provide greater energetic returns. The extraction of underground storage organs or inner bark frequently kills the parent organism outright, preventing regeneration and reducing future ecological productivity [170].

Historical famines repeatedly demonstrate how ecological desperation can trigger localised environmental collapse. Intensive bark harvesting may permanently damage forest stands; excessive collection of wild tubers can eradicate slow-reproducing species; overhunting of small fauna disrupts local trophic systems; and uncontrolled fishing or shellfish collection may destabilise aquatic ecosystems. These effects are usually compounded by concurrent pressures such as deforestation, agricultural expansion, armed conflict, and commercial exploitation [118].

Importantly, the ecological consequences of famine foraging are not simply the result of individual overexploitation but also reflect broader structural failures. Populations rarely destroy emergency ecosystems voluntarily; rather, environmental degradation arises when alternative survival options collapse. In this sense, overharvesting should be interpreted not merely as an ecological issue but also as evidence of institutional failure, inadequate relief systems, and unequal access to resources [169]. The erosion of biodiversity during famines also threatens the survival of TEK. As critical plant and animal species disappear locally, the TEK associated with them may vanish at the same time. This creates a dangerous feedback loop in which ecological degradation undermines both biological resilience and cultural memory, thereby reducing the capacity of future generations to survive subsequent crises.

## 8. Economic and Political Factors

Famines arise not simply from food shortages, but when populations lose the social, legal, and economic means to access food because of political and economic crises. Amartya Sen’s [2] entitlement approach fundamentally transformed famine analysis by arguing that starvation results from the collapse of entitlements—the mechanisms through which individuals obtain food through production, trade, labour, inheritance, or state support—rather than from aggregate food availability alone. Therefore, famine foods emerge as substitutes utilised when institutional systems fail to guarantee survival. The turn toward alternative foods, therefore, reflects a broader political-economic breakdown. These foods act as biological indicators of collapsing purchasing power, governance failure, unequal distribution systems, and structural violence. Their consumption reveals how access to food is mainly mediated by class position, political inclusion, infrastructure, military power, and global economic relations. Consequently, famine foods should not be interpreted merely as adaptive survival resources, but also as evidence of systemic exclusion within political and economic systems [172].

### 8.1. Market Collapse and Food Access

The widespread consumption of famine foods is closely associated with the disintegration of local markets and the collapse of purchasing power among vulnerable populations. During famine conditions, staple foods frequently remain physically present within markets, yet become economically inaccessible due to inflation, speculative hoarding, wage collapse, transportation disruption, or unequal commodity distribution [2,173]. In such circumstances, starvation emerges not because food vanishes entirely, but because impoverished households lose the means to acquire it.

This process disproportionately affects labourers, tenant farmers, displaced populations, and landless rural communities whose food entitlements depend heavily on unstable wages or market exchange. As grain prices rise beyond affordable limits, populations increasingly abandon conventional market systems and turn toward uncultivated biological resources available within surrounding ecosystems [14].

Moreover, economic inequality shapes access to wild resources. Wealthier households may retain livestock, stored grain, land access, or social networks that reduce dependence on dangerous substitutes, whereas poorer groups are forced earlier and more intensively into ecological foraging. Consequently, the dietary transition toward famine foods reflects a hierarchy of deprivation within famine-affected societies, revealing how class and economic status determine exposure to nutritional risk [174].

### 8.2. Policy Failures and Governance Issues

Modern famines are overwhelmingly anthropogenic events driven by political failure. Environmental shocks may trigger agricultural stress, but catastrophic starvation generally occurs only when governance systems fail—or deliberately refuse—to protect vulnerable populations. The emergence of famine foods as staple dietary resources, therefore, frequently signals profound state failure and institutional abandonment [50,175].

Historically, governments have intensified famines through policies such as forced collectivisation, land dispossession, militarised requisitioning, export prioritisation, discriminatory rationing systems, and the suppression of local coping mechanisms. In many cases, states continue exporting food commodities or maintaining elite food supplies, even while rural populations starve [162,176].

The political dimensions of famine become particularly visible during armed conflict. Warfare systematically destroys food systems by disrupting agricultural production, severing trade networks, contaminating land, displacing populations, and restricting civilian mobility. In extreme cases, starvation is weaponised intentionally as a mechanism of political control, counterinsurgency, or genocide [172]. Siege warfare, blockades, crop destruction, and aid denial deliberately force populations into ecological desperation, compelling them to survive on alternative and toxic resources or biologically hazardous substitutes.

Importantly, governance failures also shape which populations are most vulnerable to starvation. Marginalised ethnic groups, rural minorities, refugees, indigenous populations, and politically excluded communities frequently experience disproportionate famine exposure because they lack institutional protection and political representation [175].

At the same time, simplistic interpretations that attribute famines solely to authoritarianism or “bad governance” risk overlooking broader global economic structures. International debt regimes, commodity dependency, neoliberal restructuring, colonial land legacies, and unequal trade relations can significantly weaken national food sovereignty long before famine emerges [177].

### 8.3. Aid Systems and External Interventions

Rapid and equitable food assistance should prevent populations from resorting to dangerous famine foods altogether. Effective relief interventions can stabilise markets, preserve livelihoods, and interrupt the progression from food insecurity toward starvation. In practice, however, aid systems are frequently delayed, politicised, militarised, or culturally inappropriate [178].

Humanitarian access is commonly obstructed by armed groups or governments seeking to weaponise starvation strategically. Food convoys may be intercepted, manipulated for political leverage, or selectively distributed according to ethnic or ideological criteria [175].

Furthermore, externally imposed food aid often ignores local dietary systems and TEK. Standardised grain-based relief may temporarily reduce mortality while simultaneously undermining local food cultures, agricultural diversity, and TEK. Over prolonged periods, chronic dependence on imported relief commodities can weaken local production systems and accelerate the erosion of indigenous survival practices [14]. This creates a long-term vulnerability paradox. As communities become structurally dependent on external aid networks, younger generations may lose the TEK required to safely consume localised famine foods. If humanitarian systems collapse later, local populations may find themselves deprived of both institutional support and traditional survival strategies. In this sense, poorly integrated aid systems can unintentionally erode adaptive resilience while attempting to alleviate immediate suffering. Additionally, humanitarian responses often privilege emergency caloric delivery over structural reform. While food aid may reduce short-term mortality, it rarely addresses the underlying political-economic inequalities that generate famine vulnerability in the first place. Famine conditions frequently re-emerge cyclically despite repeated interventions when these are developed without land reform, conflict resolution, infrastructure investment, ecological protection, and equitable governance [175,178].

Ultimately, famine foods expose the structural violence embedded within political and economic systems. Their consumption reflects the collapse of social protections, unequal access to resources, institutional abandonment, and the failure of political systems to guarantee basic survival. When populations are forced to rely on famine foods, they become stark biomarkers of systemic exclusion—revealing how survival itself is shaped by power, inequality, and political neglect [2].

## 9. Case Studies

Famines are complex systemic breakdowns in ecological resilience, governance, and social distribution that force populations to abandon culturally normalised diets and adopt biologically marginal, often hazardous survival resources. Critically, these case studies show that famine mortality rates correlated less with absolute food scarcity than with the erosion of local autonomy, ecological access, and TEK.

### 9.1. Regional Comparisons (Asia, Africa, Europe, America)

The specific famine foods adopted during crises are heavily constrained by local ecology, climatic conditions, and inherited foraging traditions. Table 6 shows examples of famine foods used in several regions worldwide. Communities did not select these resources freely; rather, they exploited whatever biological reserves remain accessible after agricultural and market systems fail. This ecological dependence produces highly regionalised survival diets shaped by biodiversity, geography, and historical knowledge systems. These regional comparisons therefore show that famine foods are not successful substitutes for stable agriculture. Rather, they function as emergency biological stopgaps whose effectiveness depends heavily on ecological availability, labour capacity, and political conditions [14,21].

Although the historical examples presented in Table 6 represent some of the best-documented famines, more recent humanitarian crises demonstrate that famine-food utilisation remains an important survival strategy. Ethnobotanical studies conducted during the siege of Sarajevo (Bosnia and Herzegovina, 1992–1995) documented extensive use of wild edible plants, mushrooms, and lichens by urban populations isolated from conventional food supplies [6,7]. Similarly, during the Syrian conflict, communities increasingly relied on wild edible plants when agricultural production, food markets, and humanitarian supply chains were disrupted [8]. These examples illustrate that famine foods continue to play an important role in contemporary humanitarian emergencies.

Despite geographic differences, several structural patterns recur across these famine periods. First, several documented famine cases show increased reliance on foods that are less familiar, more labour-intensive, or require additional processing as conventional supplies become constrained. Second, some documented cases show increased reliance on resources obtained outside conventional food markets, including wild plants, algae, insects, bark, and other locally available materials. Third, retrospective analyses often overstate the nutritional adequacy of these resources.

While many famine foods contain valuable micronutrients and macronutrients, their actual caloric contribution is frequently constrained by seasonality, processing demands, low digestibility, and the physiological exhaustion of starving populations. The Chinese Great Famine clearly illustrates this contradiction. Although rural populations consumed bark, weeds, and kaolin clay to suppress hunger sensations, many of these substances provided negligible bioavailable nutrition and instead accelerated intestinal damage, poisoning, and metabolic collapse [53]. Similarly, the Irish reliance on marine resources during the Potato Famine has sometimes been romanticised as evidence of adaptive resilience, yet coastal harvesting remained geographically uneven and insufficient to offset the massive structural failures of colonial land policy and food distribution [180].

### 9.2. Successes and Failures in Famine Response

Historical cases indicate that the outcomes of survival strategies reflect not only the characteristics of available foods but also governance, food access, local knowledge, and the broader organisation of food systems. Many state-led interventions have failed catastrophically because they ignored cultural practices, undermined community resilience, or actively criminalised traditional coping mechanisms.

The Great Irish Famine provides a prominent example of institutional failure. British relief policy emphasised imported maize as a universal emergency ration, even though many rural Irish communities lacked both the milling infrastructure and the culinary familiarity needed to process it efficiently. Inadequately prepared maize caused severe gastrointestinal illness and produced pellagra due to niacin deficiency. Simultaneously, colonial authorities failed to meaningfully support localised marine harvesting systems that had historically buffered coastal populations during food shortages [180]. The persistence of food exports from Ireland during mass starvation further demonstrates that famine mortality emerged primarily from entitlement collapse rather than absolute food absence.

Comparable governance failures have been documented during the Great Famine of 1696–1697 in the Swedish Baltic provinces, where continued grain exports despite widespread food shortages intensified local food insecurity, illustrating how political and commercial priorities can exacerbate famine independently of agricultural production [5].

An even more severe example occurred during the Chinese Great Leap Forward. State collectivisation policies dismantled traditional subsistence systems, restricted mobility, and suppressed local food autonomy. When agricultural production collapsed, rural populations lost access not only to cultivated crops but also to the ecological fallback systems that historically mitigated shortages. The resulting consumption of bark, industrial residues, and indigestible fillers was not adaptive innovation but evidence of total systemic failure [53]. Mortality was intensified by political denial, misinformation, coercive grain procurement, and the criminalisation of independent survival practices.

In contrast, some communities achieved limited success precisely because they retained localised ecological knowledge independent of centralised institutions. During the Darfur famine, rural populations utilised inherited TEK to detoxify *Boscia senegalensis* berries despite their inherent toxicity [172]. More recent conflicts provide additional evidence that community resilience depends heavily on preserving TEK. During the siege of Sarajevo, civilians successfully supplemented severely restricted food supplies by harvesting numerous wild edible plants, mushrooms, and lichens from surrounding environments. Likewise, during the Syrian conflict, wild edible plants became increasingly important as conventional food systems deteriorated because of armed conflict, population displacement, and market disruption. These cases illustrate the practical relevance of locally transmitted ecological knowledge for identifying and preparing unconventional foods during humanitarian emergencies [6,7,8]. This strategy succeeded because communities had preserved the sophisticated processing knowledge required to render them temporarily edible. These examples show that TEK can serve as a critical resilience mechanism when formal systems collapse. However, even these “successes” require careful interpretation. Reliance on toxic wild foods often imposes substantial physiological costs, including chronic gastrointestinal disorders, micronutrient deficiencies, and long-term neurological damage [164].

### 9.3. Lessons from Localised Survival Strategies

Historical case studies indicate that access to diverse food resources can broaden the range of foods available during periods of severe scarcity. Access to uncultivated biodiversity and locally transmitted ecological knowledge has been associated with broader survival-food options in several documented contexts [14,15,22,168]. TEK can function as an accumulated body of knowledge concerning species identification, seasonality, harvesting, processing, and food preparation. Processing famine foods often requires highly specialised, complex techniques that cannot be improvised during acute starvation [182]. Without this knowledge, many biologically available resources remain effectively inedible or lethally toxic.

Yet modern industrial food systems actively erode these localised survival capacities. Agricultural modernisation, urbanisation, monoculture expansion, and the globalisation of commercial diets have accelerated the disappearance of foraging traditions and oral ecological memory [27]. As communities become increasingly dependent on centralised supply chains, they lose the practical skills necessary to exploit local biodiversity during emergencies [25].

Evidence from Bosnia and Herzegovina and Syria further indicates that these skills retain practical value in the twenty-first century. Communities possessing detailed knowledge of local edible plants were better able to diversify emergency diets when conventional food distribution systems failed. These contemporary examples reinforce the importance of documenting, preserving, and transmitting Traditional Ecological Knowledge as part of national food-security, disaster-preparedness, and climate-adaptation strategies [6,7,8].

Preserving TEK and access to uncultivated biodiversity may help maintain alternative food options during crises, although their effectiveness depends on ecological availability, social access, and food-safety considerations.

## 10. Modern Relevance

The concept of “famine foods” is undergoing a major epistemological and political transformation. Historically, these species were framed almost exclusively as desperate, inferior, or toxic survival resources consumed only during catastrophic food crises. Contemporary discourse, however, increasingly reclassifies many of these taxa as WEPs, NUS, or “forgotten foods.” This shift has occurred alongside growing scientific and policy interest in climate variability, biodiversity loss, geopolitical disruption, supply-chain vulnerability, and diversification of food systems [21,137,183].

Nevertheless, the modern rehabilitation of famine foods demands critical scrutiny. Contemporary sustainability discourse often risks romanticising species historically associated with coercion, starvation, and social trauma. Some famine-food resources may warrant investigation as components of diversified food systems, provided that nutritional adequacy, food safety, ecological sustainability, and equitable access are established [14,184].

**Figure 3:**
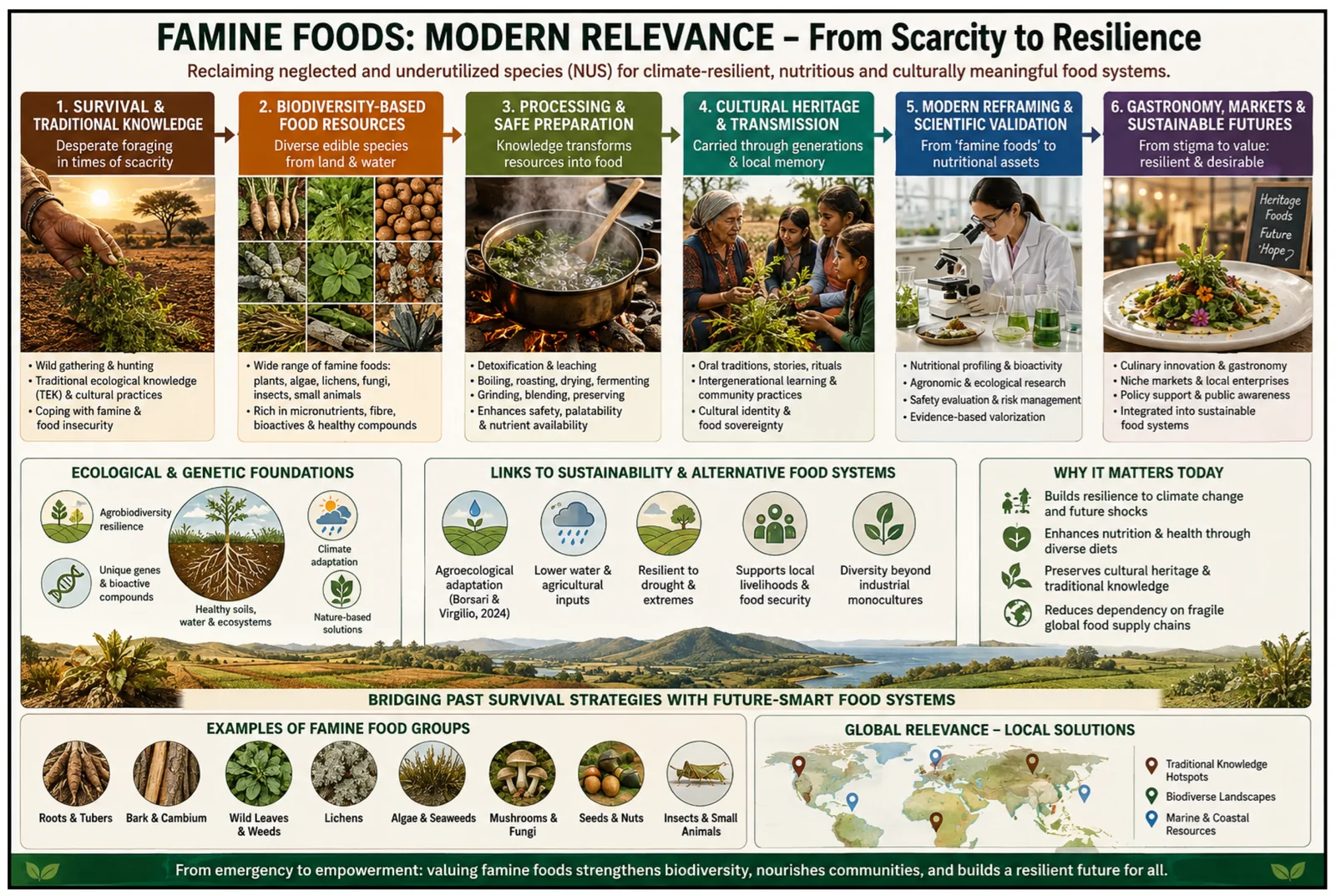
Famine Foods Reimagined: From Survival Strategy to Sustainable Food Systems [189]. Figure generated using ChatGPT (OpenAI, GPT-5.5). The authors have reviewed and edited the output and take full responsibility for the content of this publication.

Figure 3 illustrates the contemporary recontextualisation of famine foods, from emergency resources associated with scarcity and stigma to potentially valuable components of diversified and resilient food systems. It depicts a continuum linking traditional foraging and TEK with biodiversity conservation, safe processing, scientific assessment, climate adaptation, and their emerging roles in sustainable agriculture, local food systems, and gastronomy. The figure also highlights the ecological and genetic diversity that underpins this transition and emphasises the importance of integrating historical knowledge with contemporary approaches to nutrition, sustainability, and food-system resilience.

### 10.1. Famine Foods in Contemporary Food Insecurity

Famine foods are not merely historical relics; for millions of people, they contain active components of contemporary survival strategies. In drought-prone and other economically marginalised regions, particularly across parts of sub-Saharan Africa and South Asia, uncultivated wild foods continue to function as emergency nutritional buffers during seasonal hunger periods and crop failures [118]. So, in areas such as Eastern Ethiopia, rural communities routinely collect on WEPs during the “hunger season,” when household grain reserves are exhausted before new harvests mature [185].

This reality exposes a major weakness in dominant global food-security frameworks. International agricultural policy overwhelmingly prioritises intensifying a small number of globally traded staple crops while systematically overlooking localised food ecologies that sustain vulnerable populations during crises. Yet empirical studies repeatedly demonstrate that rural populations often possess extensive knowledge of dozens of wild species that can supply carbohydrates, micronutrients, and drought-resistant calories during climatic shocks [161,166].

Urbanisation, formal schooling systems, dietary globalisation, land privatisation, and the stigma historically associated with famine foods are accelerating the disappearance of intergenerational TEK [161,184,186].

At the same time, the resilience potential of famine foods is frequently overstated. Many WEPs provide only supplementary nutrition rather than complete dietary adequacy. Their availability is highly seasonal, geographically uneven, and dependent on intact ecosystems. Moreover, many species require labour-intensive processing to neutralise toxic compounds and toxins, a significant limitation for physiologically weakened populations. The practical utility of these foods, therefore, depends not merely on biological availability but on labour capacity, ecological access, and preserved TEK [14,21].

### 10.2. Links to Sustainability and Alternative Food Systems

The growing interest in NUS and WEPs is closely tied to broader critiques of industrial agriculture and global food homogenization. Modern food systems rely heavily on an extraordinarily narrow genetic base: despite thousands of historically cultivated edible species, global caloric intake is now dominated primarily by wheat, rice, and maize [187]. This concentration generates severe ecological vulnerabilities, including soil degradation, water overexploitation, reduced biodiversity, pesticide dependence, and heightened susceptibility to climate-induced crop failure [188].

In contrast, many forgotten crops evolved under marginal environmental conditions and therefore possess valuable traits under climate stress, including drought tolerance, low-input productivity, and resistance to endemic pests. Because these species frequently require fewer fertilisers, pesticides, and irrigation resources, they are increasingly promoted as sustainable alternatives capable of diversifying agricultural systems and improving resilience [189].

However, sustainability narratives surrounding forgotten foods require careful qualification. The assumption that all NUS are inherently sustainable or scalable oversimplifies major agronomic and socioeconomic constraints. Many of these species produce comparatively low yields, show inconsistent domestication traits, or remain poorly suited to large-scale mechanised agriculture. Some also contain substantial anti-nutritional compounds requiring processing infrastructure unavailable in many contexts [183,190].

Furthermore, commercialising forgotten foods introduces ethical and political concerns. As high-income markets begin to valorise previously marginalised species, there is a growing risk of biocultural appropriation, in which indigenous ecological knowledge is commodified without equitable benefit-sharing. Crops once stigmatised as “poverty foods” may become premium urban commodities while the communities that historically preserved them remain economically marginalised [184].

### 10.3. Rediscovery of “Forgotten Foods” in Gastronomy and Resilience Planning

The contemporary rediscovery of forgotten foods reflects a broader cultural shift in how societies conceptualise scarcity, sustainability, and culinary value. Species historically associated with famine and deprivation are increasingly rebranded as markers of authenticity, ecological consciousness, and gastronomic innovation. International initiatives such as the Global Manifesto on Forgotten Foods advocate reintegrating these crops into mainstream agricultural and nutritional planning as climate-resilient alternatives [187]. However, historical famine foods should not automatically be equated with sustainable or nutritionally superior alternatives. Their contemporary revalorisation requires independent assessment of nutritional quality, safety, ecological availability, processing requirements, and the historical meaning attached to their consumption.

In resilience planning, governments and research institutions are actively investigating the adaptive potential of neglected crops under future climate scenarios. Species previously dismissed as weeds or famine resources—such as spider plant (*Cleome gynandra*)—are now being evaluated for their nutritional density, drought tolerance, and ecological adaptability in regions vulnerable to declining staple yields [191]. Simultaneously, elite gastronomy and urban food culture have begun to appropriate foraged and historically famine foods as symbols of environmental sophistication and culinary heritage. Wild greens, edible weeds, sea vegetables, and historical survival staples increasingly appear in high-end restaurants, farmers’ markets, and “slow food” movements [189]. This process partially de-stigmatises species once associated with hunger and may generate new economic opportunities for smallholder producers.

Yet this culinary rehabilitation contains significant contradictions. Transforming famine foods into fashionable commodities can obscure the historical suffering associated with their consumption. Foods once eaten under coercive starvation conditions may become aestheticised as luxury experiences detached from their traumatic contexts. Affluent sustainability discourse may therefore sanitise famine history while neglecting the structural inequalities that originally forced populations to rely on these resources [2,184]. Moreover, gastronomic interest alone does not guarantee meaningful transformation of the food system. Many forgotten foods remain niche products inaccessible to populations most vulnerable to food insecurity. Without structural reforms addressing poverty, land access, ecological degradation, and agricultural inequality, the symbolic celebration of forgotten foods risks remaining largely performative [177,183].

## 11. Methodological Approaches

Studying famine foods requires an inherently interdisciplinary methodological framework because starvation is simultaneously a biological, ecological, political, and cultural phenomenon. No single analytical approach can adequately reconstruct how populations survived food-system collapse, how knowledge regarding toxic wild resources was transmitted, or how emergency diets interacted with broader structures of power and environmental change. Consequently, modern famine-food research integrates archaeobotany, archival analysis, ethnography, nutritional biochemistry, toxicology, climatology, and ecological modelling [50,192].

At the same time, methodological limitations remain substantial. Much of the available evidence is fragmentary, geographically uneven, and shaped by survivor bias. Starving populations rarely leave systematic dietary records, while many emergency foods leave weak archaeological signatures. Furthermore, contemporary scholarship sometimes risks projecting modern nutritional or sustainability frameworks onto historical populations whose consumption choices were driven primarily by desperation rather than informed dietary optimisation. Methodological rigour, therefore, requires constant critical evaluation of evidence quality, interpretive assumptions, and disciplinary blind spots [137,162].

### 11.1. Archaeobotany and Historical Records

Reconstructing famine diets in antiquity and historical periods depends heavily on archaeobotanical evidence and documentary archives. The preservation of famine foods in the archaeological record is inconsistent and often poor because many famine foods consist of wild plants, bark tissues, opportunistic weeds, insects, or highly perishable resources.

Table 7 details the primary methodologies bridging archaeobotany and historical analysis. As a result, famine-related dietary reconstruction is methodologically difficult and frequently inferential rather than definitive. Archaeobotanical analysis typically relies on flotation techniques to recover carbonised macrobotanical remains such as seeds, nutshells, and plant fragments from archaeological sediments [192,193]. Some researchers also analyse microbotanical remains—including phytoliths, starch grains, and pollen—from grinding tools, cooking vessels, and dental calculus to identify otherwise invisible dietary components [194].

These approaches have allowed scholars to detect abrupt dietary shifts, such as increased reliance on wild grasses, root crops, or heavily fibrous plant tissues associated with food stress. However, interpreting these remains is highly complex. Many famine foods overlap with normal subsistence resources, making it difficult to distinguish routine foraging from emergency consumption. Furthermore, preservation bias disproportionately favours charred or mineralised materials while underrepresenting soft tissues, leaves, seaweed, and many animal resources. Consequently, archaeobotanical evidence alone rarely proves famine conditions conclusively.

Historical records, providing textual documentation of food crises, can partially compensate for these limitations. Historical records, providing textual documentation of food crises, can partially compensate for these limitations. Administrative reports, merchant ledgers, taxation records, relief documents, personal diaries, and parish registries can reveal fluctuations in food prices, mortality rates, crop failures, and descriptions of emergency foods [50]. When integrated with archaeobotanical evidence, these records can help reconstruct chronological advances of famine progression and documented survival strategies.

Yet archival evidence also contains major distortions. State documents frequently minimise famine severity for political reasons, while elite chroniclers often ignore or stigmatise the diets of poor populations. Many survival foods consumed by marginalised groups were considered socially degrading, and therefore, they went underreported. Consequently, historical silence surrounding famine foods may reflect social prejudice as much as actual dietary absence [50,162].

### 11.2. Ethnographic Fieldwork

Ethnographic fieldwork is central to understanding the cultural, symbolic, and practical dimensions of famine foods that archaeological remains alone cannot capture. This approach documents how living communities identify edible species, process toxic resources, classify emergency foods, and transmit survival knowledge across generations. In many cases, community elders serve as the final repositories of oral histories related to historical famines and localised survival practices [182].

Participant observation and semi-structured interviews allow ethnographers to document harvesting techniques, seasonal ecological patterns, detoxification practices, and food taboos. Methods such as free-listing, pile sorting, and cultural domain analysis help researchers map how communities cognitively organise local flora and fauna into categories such as staple foods, medicinal species, famine foods, or taboo organisms [197].

Ethnographic approaches are particularly valuable for recording complex processing systems required to neutralise toxins in famine foods. Many detoxification methods previously described represent highly specialised knowledge accumulated over centuries of experimentation. Without ethnographic preservation, much of this information risks disappearing due to rapid social and ecological change [15,161].

Nevertheless, ethnographic methodologies face important limitations. Oral histories are shaped by memory distortion, trauma, stigma, and retrospective reinterpretation. Survivors may minimise, conceal, or selectively remember famine-related experiences, especially when consuming certain foods carried shame or social degradation. Additionally, modern ethnographic observation cannot automatically be projected backwards into ancient contexts. Contemporary foraging systems may differ substantially from historical survival strategies due to environmental change, market integration, and cultural transformation [50,192].

There is also a growing ethical concern regarding the extraction and commodification of indigenous knowledge. Researchers documenting TEK must confront issues of intellectual ownership, consent, and equitable benefit-sharing, particularly as TEK increasingly intersects with commercial agriculture, pharmaceuticals, and sustainability initiatives [43].

### 11.3. Nutritional Analysis Techniques

Nutritional biochemistry provides the quantitative foundation for evaluating the actual physiological value of famine foods. Historical records may document what populations consumed, but biochemical analysis determines whether those resources provided sufficient nutritional support or merely psychologically delayed starvation through gastric bulk.

Standard proximate analysis—often following AOAC protocols—measures moisture, protein, lipid, ash, carbohydrate, and fibre content [198]. Advanced techniques such as High-Performance Liquid Chromatography (HPLC) and Gas Chromatography (GC) coupled to several detectors, emphasising Mass Spectrometry (MS), enable precise quantification of amino acids, fatty acids, vitamins, phytochemicals, and minerals.

Toxicological analysis is equally essential because many famine foods contain potent anti-nutritional compounds or toxic secondary metabolites. Analytical methods are used to quantify cyanogenic glycosides, oxalates, alkaloids, tannins, lichen acids, and other harmful compounds to evaluate the risks associated with emergency consumption [36]. These analyses also allow scientists to experimentally assess whether traditional processing methods effectively reduce toxicity.

However, laboratory-based nutritional analysis has major interpretive constraints. Chemical composition alone does not necessarily reflect real nutritional outcomes during famine conditions. Bioavailability, digestibility, pathogen exposure, metabolic stress, and caloric expenditure associated with harvesting and processing significantly alter the net survival value of foods. Some species with impressive nutrient profiles may provide little practical benefit if toxins inhibit absorption or if preparation demands exceed available labour capacity [14,137].

Furthermore, contemporary nutritional frameworks may unintentionally depoliticise famine by reducing survival foods to biochemical metrics while neglecting the structural violence that forced populations to consume them. A nutritionally “valuable” famine food may still represent evidence of catastrophic social collapse [4,175].

### 11.4. Interdisciplinary Approaches

The most sophisticated contemporary research on famine foods increasingly relies on interdisciplinary synthesis because isolated biological or cultural variables cannot adequately explain starvation. Interdisciplinary methodologies allow researchers to integrate ecological data, nutritional science, historical evidence, climate modelling, and ethnographic knowledge into a more comprehensive understanding of human survival strategies.

One major interdisciplinary framework is ethnoarchaeology, which uses contemporary observations of traditional food processing to interpret archaeological residues and ancient subsistence systems [199]. Similarly, nutritional ethnobiology combines ethnographic documentation with biochemical analysis to scientifically evaluate the nutritional and toxicological validity of TEK [198].

Climatology and Geographic Information Systems (GIS) have also become increasingly important. Researchers now combine paleoclimate reconstruction, dendrochronology, pollen analysis, and species-distribution mapping to model how environmental fluctuations affected the availability of famine foods across historical landscapes. These tools help estimate ecological carrying capacity, migration pressures, and the resilience of uncultivated food systems during climatic shocks [168,192].

Yet even interdisciplinary approaches face significant challenges. Integrating evidence across disciplines often introduces incompatible assumptions, scales of analysis, and methodological standards. Quantitative nutritional data may conflict with culturally embedded interpretations of food value, while ecological modelling can oversimplify social complexity and political causation. There is also a persistent risk of deterministic reasoning that portrays famine adaptation as an inevitable ecological process while minimising the role of governance failure, colonialism, warfare, and economic exploitation [2,137,175].

## 12. Concluding Remarks

### 12.1. Summary of Insights

Famine foods reflect the extreme conditions under which conventional food systems fail. Environmental stress, conflict, economic disruption, governance failures, and unequal food access shape their use. Historical and contemporary cases show that wild and unconventional foods can provide emergency resources during severe scarcity, but their use should not be equated with nutritional adequacy or systemic resilience.

Famine foods encompass diverse plant, animal, and processed resources with highly variable nutritional and toxicological profiles. People have used uncultivated plants, NUS, unconventional animals, and marginal food substitutes during food crises. Their nutritional value depends strongly on species, edible part, preparation, and consumption conditions. Dry-weight composition can also overestimate their practical nutritional contribution when foods are consumed fresh or incompletely processed.

Many famine foods also contain toxic compounds, antinutritional factors, allergens, or infectious hazards. Traditional processing and preparation can reduce some risks, but inadequate preparation may increase exposure during desperate circumstances. Famine-food consumption should thus be understood as an emergency response rather than evidence that these resources are universally safe or nutritionally sufficient.

Traditional ecological knowledge (TEK) is central to identifying, collecting, processing, and safely consuming many famine foods. Ethnobotanical and ethnozoological evidence shows that cultural knowledge strongly shapes which resources people accept or avoid. Displacement, conflict, and social disruption may weaken this knowledge and increase risks.

Famine-food use is also socially and culturally structured. Access to land, labour, markets, social networks, and transmitted knowledge can determine who can exploit these resources and under what conditions. Similar environments may support different food practices because cultural norms, historical experience, and social values shape perceptions of edibility and acceptability. Biodiversity can provide an important survival buffer, yet excessive extraction during crises may also threaten the ecosystems on which this buffer depends.

Famine-food research should examine how ecological conditions, food access, knowledge, and social context interact during severe scarcity. Integrating archaeobotanical, nutritional, ethnographic, historical, and ecological evidence can improve interpretation of emergency diets and resource use. Combining material evidence with documentary and oral-history sources may also clarify how famine-food practices changed across time and environments.

### 12.2. Future Directions

The perception of famine foods is changing. Increasingly framed as “forgotten foods,” climate-resilient crops, or agroecological resources, they now attract interest in sustainability, biodiversity, and alternative food systems. Their growing gastronomic and commercial appeal may support biodiversity and local livelihoods, but it can also detach these foods from the deprivation and coercion that shaped their historical use. This distinction should remain central to future research and public communication.

Future studies should document contemporary famine-food use in conflict zones, seasonal shortages, and humanitarian emergencies. Ethnobotanical surveys should be integrated with nutritional, toxicological, and public-health assessments. Particular attention is needed for chronic toxicity, nutrient bioavailability, allergenicity, and the safety of traditional processing methods. Standardised biochemical analyses could help establish preparation requirements and evaluate longer-term health effects.

Research should also assess the potential contribution of WEPs and NUS to diversified food systems. Their tolerance of environmental stress may provide useful traits for climate-resilient agriculture and breeding programmes. However, domestication and commercialisation should avoid genetic erosion, ecological simplification, and the loss of local knowledge. Ecological modelling could further identify species distribution, regeneration capacity, and vulnerability under projected climate scenarios.

The preservation of TEK is equally important. Younger generations may have less access to the knowledge required for identifying, processing, and safely consuming wild resources. Ethical documentation should recognise knowledge-holding communities as active contributors rather than passive sources of information.

Urban and peri-urban contexts also require greater attention. Modern food insecurity increasingly affects populations dependent on vulnerable logistical networks. Urban foraging, decentralised production, alternative food networks, and locally transmitted ecological knowledge may provide useful areas for investigation.

Overall, famine foods should be understood as heterogeneous resources whose value depends on ecological availability, cultural knowledge, processing, food safety, and access to conventional foods. Their nutritional potential should not be separated from toxicological constraints and practical requirements. Further interdisciplinary research is needed before individual famine foods are promoted as sustainable or resilient alternatives.

## Figures and Tables

**Table 1:** General classification of famine foods.

Category	Examples	Primary Function	References
Wild Vegetables	Wild tubers, pine bark, lichens, fungi, and forest fruits.	Caloric buffer and micronutrient source.	[13,18,23,27]
Unconventional Animals	Rodents, spiders (e.g., orb-weavers), and “pest” species.	High-quality protein and amino acid supply.	[28,29]
Processed Substitutes	Acorn flour bread, seaweed-based products, and bone meal.	Grain substitution and volume expansion.	[13,30]

**Table 2:** Cultural Classification of Famine Resources.

Category	Cultural Perception	Examples	Reference
“Real” Food	Civilised, culturally sanctioned, a source of dignity.	Traditional staples (grains, livestock).	[34]
“Bridge” Substances	Liminal; neither meal nor non-food; tolerated in crisis.	Snacks, wild fruits, pantawid-gutom.	[34]
“Non-food”/Famine Food	Desperate, stigmatised, often considered “unfit.”	Tree bark, lichens, unconventional proteins.	[13]

**Table 3:** Classification, Processing, and Selected Examples of Wild and Unconventional Famine Foods.

Category	Selected Botanical/Biological Examples	Primary Nutritional Value/Function	Processing/Preparation	References
Roots/underground storage organs (USOs)	Wild yams (*Dioscorea* spp.); bitter yam (*D. dumetorum*); wild yam (*D. hispida*); water-arum roots (*Calla palustris*)	Mainly starch and carbohydrates; important emergency energy source	Depending on species: peeling, slicing, soaking, repeated washing, boiling/steaming, drying, leaching; some species require prolonged detoxification	[13,59,60]
Tubers/rhizomes	Potato tubers and peels (*Solanum tuberosum*); chicory root (*Cichorium intybus*); cattail rhizomes (*Typha* spp.); wild yam tubers	Starch and carbohydrates; some roots provide soluble dietary fibre	Washing, peeling, boiling, roasting or drying; roasting and grinding for some roots	[18,59]
Bulbs/ornamental underground organs	Tulip bulbs (*Tulipa* spp.); dahlia tubers (*Dahlia* spp.); other ornamental bulbs documented during the Dutch Hunger Winter	Carbohydrate/energy source when conventional crops were unavailable	Peeling, cooking, processing into soups or other preparations; some bulbs require careful selection because of toxicity	[18]
Tree inner bark/cambium	Scots pine (*Pinus sylvestris*); birch (*Betula* spp.); spruce (*Picea* spp.); ash (*Fraxinus* spp.); elm (*Ulmus* spp.)	Carbohydrates, fibre; emergency flour extender	Preferential harvesting during suitable seasons; drying, roasting/kiln-drying, grinding and mixing with cereal flour; porridge preparation	[13]
Tree bark: tropical/Indigenous examples	Maya food plants including tree resources from Fabaceae and Malvaceae; other locally available tree tissues	Carbohydrates and structural plant material; emergency food resource	Localised harvesting and processing; preparation varied according to species and cultural knowledge	[61]
Wild leafy vegetables	Stinging nettle (*Urtica dioica*); amaranth (*Amaranthus* spp.); sorrel (*Rumex* spp.); chickweed (*Stellaria media*); dandelion (*Taraxacum officinale*)	Vitamins A and C, minerals, fibre and some protein; micronutrient supplementation	Usually washing and cooking/blanching; preparation depends on species and potential anti-nutritional compounds	[18,26]
Wild leaves/foliage from cultivated or fodder plants	Radish foliage (*Raphanus sativus*); carrot leaves (*Daucus carota*); turnip greens (*Brassica rapa*); Brussels-sprout leaves (*Brassica oleracea*)	Vitamins, minerals and fibre; dietary supplementation during severe shortages	Washing, boiling or incorporation into soups and stews	[18]
Wild fruits/berries	Blackberry (*Rubus* spp.); rose hips (*Rosa* spp.); wild berries; sloes (*Prunus spinosa*); wild fruits documented in European famine contexts	Sugars, carbohydrates, vitamin C and phenolics; fresh food and micronutrient source	Usually eaten fresh, dried, cooked or incorporated into drinks/preserves	[7,18,26]
Wild seeds/nuts	Beechnuts (*Fagus sylvatica*); walnuts (*Juglans regia*); sweet chestnuts (*Castanea sativa*); hazelnuts (*Corylus* spp.)	Lipids, protein and energy; some provide substantial essential fatty acids	Drying, roasting, grinding; some seeds require treatment to reduce bitterness or toxic compounds	[18,26]
Wild cereals/grass seeds	Wild grasses; couch grass (*Elymus repens*); grain ears and chaff used as cereal extenders	Carbohydrates, starch and fibre; grain substitution/extension	Harvesting, drying, threshing, windowing and grinding; incorporation into flour or porridge	[13,26]
Lichens	Iceland moss (*Cetraria islandica*); reindeer lichens (*Cladonia* spp.); rock tripe (*Umbilicaria* spp.)	Complex polysaccharides, mainly lichenin/lichenan; emergency carbohydrate source	Extended soaking and boiling; alkaline/ash treatment or repeated washing may be used to reduce bitterness and irritant compounds	[62,63,64]
Edible fungi	*Boletus* spp.; *Lactarius deliciosus*; *Cantharellus* spp.; other wild mushrooms documented in wartime famine	Protein, minerals, B vitamins and some lipids; mainly dietary supplementation	Correct identification is essential; washing and cooking; some species require specific preparation	[6,18]
Marine macroalgae/seaweeds	Dulse (*Palmaria palmata*); carrageen moss (*Chondrus crispus*); laver (*Porphyra* spp.); wracks (*Fucus*, *Ascophyllum* spp.)	Minerals, iodine, dietary fibre, polysaccharides and some protein and PUFA	Washing/rinsing to remove sand and epiphytes; drying, boiling or incorporation into soups/bread depending on species	[65,66]
Aquatic/freshwater wild plants	Watercress (*Nasturtium officinale*); water-arum roots (*Calla palustris*); locally available aquatic edible plants	Vitamins, minerals and carbohydrates depend on organ	Thorough washing; boiling/cooking; some species require detoxification	[13,26]
Famine bread extenders/processed plant substitutes	Bark flour, straw, chaff, bran, potato peel, vetch/pea flour, wild-seed flour	Mainly bulk, carbohydrates and fibre; nutritional contribution highly variable	Drying, milling and incorporation into bread or porridge; some provide little bioavailable energy	[13,18,62]
Processed unconventional plant foods	Sugar beet (*Beta vulgaris*); tulip bulbs (*Tulipa* spp.); potato peels (*Solanum tuberosum*) during the Dutch Hunger Winter	Carbohydrate and limited energy when conventional staples were unavailable	Cooking and incorporation into soup-kitchen foods; preparation depended on the material	[18]

**Table 4:** Comparison of the Main Famine Bread Substitutes.

Substitute Ingredient	Botanical Source	Primary Nutritional Value	Processing Requirements	Physiological or Nutritional Considerations	Reference
Pine cambium	*Pinus* spp., including *P. sylvestris*	Carbohydrates, dietary fibre, vitamin C, minerals	Inner bark harvesting, drying, and milling; incorporation into flour or bread	Provided a complementary source of carbohydrates and micronutrients during seasonal scarcity; high fibre content may limit digestibility	[70]
Couch grass rhizome	*Elymus repens*	Starch and soluble carbohydrates	Digging, washing, drying, grinding; used in gruels, soups, or bread	Traditionally used as a food during scarcity; preparation enabled incorporation into staple foods	[26,73]
Acorn flour	*Quercus* spp.	Starch, lipids, protein, fibre, minerals	Shelling, drying or roasting, prolonged soaking/leaching, grinding	Nutritionally substantial but tannin-rich; debittering reduces tannins and improves suitability for food use	[74,75,76]
Wild amaranth seeds	*Amaranthus* spp.	Protein, minerals, and other nutrients	Collection, threshing, windowing, drying, milling	Documented as famine foods when preferred foods were unavailable; nutritional contribution varies with species and seed preparation	[73,77]
Sawdust/wood-derived pulp	Various wood species	Very limited nutritional contribution	Processing into pulp or incorporation into emergency bread mixtures	Used under extreme scarcity primarily as a bulking or flour-extending material rather than as a nutritionally equivalent food	[68,69]

**Table 5:** Classification and Nutritional Viability of Unconventional Fauna.

Category	Biological Examples	Primary Nutritional Value	Required Preparation/Processing	Reference
Insects (Swarming)	Desert locust (*Schistocerca gregaria*)	High protein, essential fatty acids, Zn, Fe	Catching in nets/trenches; roasting, boiling, or sun-drying into powder.	[80,81]
Insects (Larval/Soil)	Termites (*Isoptera*), Beetle grubs	Exceptionally high lipids (fats), protein	Foraging from mounds or dead wood; typically roasted or fried in their own fat.	[80]
Synanthropic Rodents	Brown rat (*Rattus norvegicus*)	Complete proteins, fats	Trap, boil intensively, or roast to destroy dangerous zoonotic pathogens.	[79]
Companion Animals	Domestic dog (*Canis familiaris*), Cat (*Felis catus*)	Complete animal protein, fats	Standard butchery; historically, it was eaten only after all livestock and game were depleted.	[79]
Processed Byproducts	Leather (shoes, belts, saddles)	Trace collagen/gelatin (very low caloric value)	Intensive boiling for 12–24 h to soften tough fibres and extract hide-glue/jelly.	[53]

**Table 6:** Regional Comparison of Selected Historical Famine Foods.

Region	Historical Famine Context	Primary Famine Foods Utilised	References
Africa	Sudan (Darfur Famine, 1984–1985)	*Boscia senegalensis* (Mukheit) berries, wild grasses, locusts, baobab leaves	[172,179]
Asia	China (Great Leap Forward, 1958–1962); Syria Syrian Conflict (2011–present)	Elm bark, chaff, weeds, roots, water hyacinths, and in extreme cases, kaolin clay	[10,53]
Europe	Ireland (Potato Famine, 1845–1849); Netherlands (Hunger Winter, 1944–1945); Bosnia (Bosnian War, 1992–1995)	Marine algae (dulse, kelp), limpets, nettles (Ireland); Tulip bulbs, sugar beets (Netherlands)	[180,181]
Americas	Mesoamerica and North America (Pre-colonial droughts and colonial displacement)	Sego lily bulbs, mesquite pods, ramon nuts, processed leather, and tree inner bark	[77]

**Table 7:** Methodologies in Archaeobotany and Historical Analysis of Famine Foods.

Methodological Approach	Analytical Focus	Application/Context	Key References
Macrobotanical Analysis	Identification of carbonised seeds, nuts, and chaff via flotation.	Identifying the sudden inclusion of uncultivated weeds or toxic legumes (e.g., *Lathyrus sativus*) in ancient hearths.	[192,193].
Microbotanical Analysis	Analysis of phytoliths, starch grains, and pollen grains.	Reconstructing the consumption of starchy root famine foods (e.g., Sego lily) from tool residue or dental calculus.	[194]
Coprolite Analysis	Microscopic and chemical analysis of desiccated/mineralised faeces.	Providing direct archaeological evidence of ingested food materials, although preservation and identification constraints may limit interpretation of complete dietary patterns.	[195]
Archival Documentation	Review of parish registries, relief commission reports, and diaries.	Correlating crop failure data (e.g., potato blight) with the documented foraging of marine algae and nettles.	[10,180]
Isotopic Analysis	Stable isotope ratios (Carbon, Nitrogen) in human skeletal remains.	Detecting rapid shifts in dietary baselines (e.g., from C3 crops to marine resources) immediately preceding death.	[196]

## Data Availability

No new data were created or analysed in this study. Data sharing is not applicable to this article.

## Supplementary Materials

The following supporting information can be downloaded at: https://www.mdpi.com/article/10.3390/foods15203602/s1, Table S1: Nutritional composition (g, mg or kcal/100 g) of selected vegetal-origin famine foods; Table S2: Nutritional composition (g, mg or kcal/100 g) of animal-origin famine foods; Table S3: Toxic and anti-nutritional compounds in famine foods, effect on health and mitigation treatment.

## Abbreviations

The following abbreviations are used in this manuscript:

ALA	Alpha-linolenic acid
AOAC	Association of Official Analytical Collaboration
BMAA	β-N-methylamino-L-alanine
β-ODAP	β-N-oxalyl-L-α,β-diaminopropionic acid
Ca	Calcium
CH	Carbohydrates
Cu	Copper
DW	Dry weight
EPA	Eicosapentaenoic acid
FAO	Food and Agriculture Organization
Fe	Iron
FW	Fresh weight
G	Grams
GC	Gas chromatography
GI	Gastrointestinal
GIS	Geographic Information Systems
HPLC	High-performance liquid chromatography
I	Iodine
JSTOR	Journal Storage
K	Potassium
Kcal	Kilocalories
LA	Linoleic acid
Mg	Magnesium
MS	Mass Spectrometry detector
Mg	milligrams
Na	Sodium
NUS	Neglected and underutilised species
OA	OA acid
P	Phophorus
PSTs	Paralytic Shellfish Toxins
SDA	Stearidonic acid
TEK	Traditional ecological knowledge
μg	Micrograms
USO	Underground Storage Organs
WEFs	Wild edible fruits
WHO	World Health Organisation
Zn	Zinc
