# Supplementary material for: Famine Foods: A Structured Narrative Review of Nutritional, Toxicological, Ecological, and Socio-Cultural Perspectives
Source: Foods. 2026 Oct 10;15(20):3602. doi: 10.3390/foods15203602 (PMC13703261; doi:10.3390/foods15203602)
Supplement: Supplementary file 1 [file foods-15-03602-s001.zip › Supplementary Table S1.pdf]

Supplementary Table S1. Nutritional composition (g, mg or kcal/100 g) of selected vegetal-origin famine foods

Species (Common Name)	Moisture (g)	Energy (kcal)	Protein (g)	Total CH (g)	Lipids (g)	Omega (n) -3, 6, 9 Profile (% of Total Fatty Acids)	Ash (g)	Vit C (mg)	Main Mineral Elements	Key Phytochemicals, Tocols & Carotenes	Reference
Algae											
Ulva lactuca (Sea Lettuce) FW	81.2	45	2.5	8.0	0.1	n-3: ~7% (ALA, SDA)<br>n-6: ~9% (LA)<br>n-9: ~37% (OA)	5.5	41.0	Na, Ca, P, Mg, Mg	Ulvans, phenolics, flavonoids, γ-tocotrienol chlorophylls	[86-88]
Palmaria palmata (Dulse) FW	85.0	65	2.5	8.0	0.50	n-3: ~50.0% (EPA)<br>n-6: ~2% (AA)<br>n-9: ~3% (OA)	4.8	62	Na, Ca, K, Mg, Mg, Cu, I, Zn	Phycoerythrin, phlorotannins	[66,89,90]
Lichens											
Cetraria islandica (Iceland Moss) DW	12.0	~250	1.5	75.0	0.20	n-3: ~10% (ALA)<br>n-6: ~40% (LA)<br>n-9: ~30% (OA)	2.5	Traces	Ca, K, Fe	Lichenin, isolichenin, cetraric acid	[63,91]
Fungi											
Boletus edulis (Porcini) FW	88.0	35	3.9	4.1	0.50	n-3: <2%<br>n-6: ~45% (LA)<br>n-9: ~30% (OA)	1.1	4.3	K, P, Mg, Se	Ergosterol, phenolic acids	[92]
Lactarius deliciosus (Saffron Milk Cap) FW	89.5	34	2.1	5.5	0.50	n-3: <1%<br>n-6: ~15% (LA)<br>n-9: ~40% (OA)	0.9	3.1	K, P, Ca	Sesquiterpenes, tocopherols	[92]
Wild Greens											
Amaranthus viridis (Slender Amaranth) FW	87.5	35	3.2	6.5	0.19	n-3: ~40% (ALA)<br>n-6: ~20% (LA)<br>n-9: ~10% (OA)	2.5	45.0	Ca, K, Mg, Fe	Carotenes (high DW fraction), betalains	[93,94]
Chenopodium album (Lamb's Quarters) FW	85.0	40	3.5	8.2	0.33	n-3: ~45% (ALA)<br>n-6: ~20% (LA)<br>n-9: ~10% (OA)	2.8	65.0	Ca, K, Mg, Fe	Carotenes: 30.5 mg/100g DW<br>Tocopherols 2.1 mg/100 g FW<br>Flavonoids: quercetin and luteolin	[93,95-97]

Portulaca oleracea (Purslane) FW	92.5	16	1.3	3.4	0.2	n-3: ~40% (ALA)<br>n-6: ~25% (LA)<br>n-9: ~10% (OA)	1.6	21.0	K, Mg, Ca, P, Fe	Phenolics and Flavonoids: Caffeic acid glucoside, catechin, quercetin; α-tocopherol; β-carotene	[93,98,99]
Sonchus oleraceus (Common Sowthistle) FW	87.2	26	3.2	5.1	0.70	n-3: ~45% (ALA)<br>n-6: ~25% (LA)<br>n-9: ~5% (OA)	3.0	78	K, Ca, Mg, P, Fe	-Flavonoids (luteolin, apigenin, quercetin, and their glucosides) and phenolic acids (chlorogenic and cichoric acid<br>-Sesquiterpene lactones<br>-α-tocopherol<br>-β-carotene	[93,100]
Crithmum maritimum (Sea Fennel) FW	86.8	62	2.2	6.7	0.43	n-3: ~20% (ALA)<br>n-6: ~20% (LA)<br>n-9: ~30% (OA)	3.5	45.0	Na, K, Ca, Mg, P, Mn, Zn	Carotenes, Flavonoids, apiol, essential oils	[101]
Wild Fruits											
Arbutus unedo (Strawberry Tree Fruit) FW	56.0	165	0.9	40.0	0.50	n-3: ~30% (ALA)<br>n-6: ~20% (LA)<br>n-9: ~20% (OA)	0.9	180.0	K, Ca, P,Fe, Zn	Phenolics: proanthocyanidins, gallic acid, ellagic acid<br>-Anthocyanins<br>-α-tocopherol<br>-Violaxanthin	[102,103]
Rosa canina (Rosehip) FW	50.0	162	1.5	39.0	1.0	n-3: ~20% (ALA)<br>n-6: ~50% (LA)<br>n-9: ~15% (OA)	1.8	450.0	K, Ca, Mg, P	-Galactolipids<br>-Flavonoids (quercetin, rutin, catechin)<br>-α- γ-tocopherol<br>-Lycopene, rubixanthin, β-carotene	[103-105]
Other organs											
Inner Bark of *Pinus sylvestris* (Scots pine) DW	55.0	300	2.5	80.9, most fibres; starch ~8.0	6.5	n-3: ~5% (ALA)<br>n-6: ~10% (LA)<br>n-6: ~15% (Pinoleic acid)<br>n-9: ~40% (OA)	3.0	traces	Ca, K, Mg, Fe, Zn	Oligomeric proanthocyanidins, catechins, taxifolin, and phenolic acids (ferulic and vanillic acid).	[70,106,107]

Seeds of Sycamore tree (*Acer pseudoplatanus*) DW	15.0	365	15.0	55.0	10.0	n-3: ~5% (ALA)<br>n-6: ~50% (LA)<br>n-9: ~30% (OA)	5.0	traces	K, Ca, Mg, P	Polyphenols (gallic acid, chlorogenic acid, and catechins), hypoglycin A, tocopherols, β-sitosterol	[108,109]
Root of Wild Carrot (*Daucus carota*) (Queen Anne's Lace) FW	85.0	45.0	1.5	12.0 Mostly fiber	0.2	Traces	1.5	6.0	K, Ca, P, Mg, Na	Polyacetylenes: falcarinol and falcarindiol.	[109,110]
Bulbs of Sego Lily (*Calochortus nuttallii*) DW	70.0	360	5.0	80.0 Mainly starch	1.0	Traces	4.5	trace	K, Ca, P, Mg	saponins	[111,112]
Cultured plants											
Lettuce (*Lactuca sativa*) var. Romaine	95.0	16	1.3	3.3 (2.0 g of fibre)	0.2	n-3: ~55% (ALA)<br>n-6: ~20% (LA)<br>n-9: ~10% (OA)	0.7	14.0	K, Ca, P, Mg	-Sesquiterpene lactones (lactucin and lactucopicrin).<br>-Phenolics (chlorogenic acid and quercetin).<br>-α-and γ-tocopherols, -Carotenes: β-caroten(and lutein	[113]
