# Supplementary material for: Famine Foods: A Structured Narrative Review of Nutritional, Toxicological, Ecological, and Socio-Cultural Perspectives
Source: Foods. 2026 Oct 10;15(20):3602. doi: 10.3390/foods15203602 (PMC13703261; doi:10.3390/foods15203602)
Supplement: Supplementary file 1 [file foods-15-03602-s001.zip › Supplementary Table S2.pdf]

Supplementary Table S2. Nutritional composition (g, mg or kcal/100g) of animal-origin famine foods

Food Source	Moisture (g)	Energy (kcal)	Protein (g)	Lipids (g)	Omega (n) -3, 6, 9 Profile (% of Total Fatty Acids)	Ash (g)	Main Minerals	Tocols (mg/100 g FW)	Zoochemicals	Reference
Rodents (small mammals)	60–70	150–250	18–22	8–15	n-3: ~1-5% (ALA)<br>n-6: ~15-30% (LA)<br>n-9: ~30-50% (OA)	1–2	Fe, Zn, P, K	1–3	Bioactive dipeptides: carnosine and anserine.	[22,28]
Locusts / Grasshoppers	~60	150–200	20–25	10–20	n-3: ~1-3% (ALA)<br>n-6: ~30-50% (LA)<br>n-9: ~20-40% (OA)	3–6	Fe, Ca, Mg, Zn	5–15	Chitin	[22,122-124]
Termites	50–65	250–450	20–35	15–30	n-3: ~0.5-2% (ALA)<br>n-6: ~20-40% (LA)<br>n-9: ~30-50% (OA)	2–4	Fe, Ca, P, Mg	5–10	Chitin	[22,125]
Spiders (arachnids)	65–75	120–180	15–20	5–10	n-3: ~1-2% (ALA)<br>n-6: ~15-35% (LA)<br>n-9: ~25-45% (OA)	1–2	Fe, Zn	Low	Chitin<br>Raw spider venom is rich in neurotoxic peptides (μ-theraphotoxin)	[122,126]
Molluscs (snails, bivalves)	75–85	70–120	12–18	1–5	n-3: ~20-40% (ALA)<br>n-6: ~6-15% (LA)<br>n-9: ~10-25% (OA)	1–2	Fe, Ca, Se, Zn	1–5	Taurine, plasmalogens	[127]
Small fish (whole)	65–75	120–200	18–22	2–12	n-3: ~15-35% (ALA)<br>n-6: ~5-15% (LA)<br>n-9: ~20-40% (OA)	1–3	Ca, P, Fe, Zn	2–8	Coenzyme Q10, taurine, retinol, and cholecalciferol	[119,120]
Frogs (amphibians)	75–80	80–120	16–20	0.5–5	n-3: ~5-15% (ALA)<br>n-6: ~10-25% (LA)<br>n-9: ~20-40% (OA)	1–1.5	P, K, Fe	1–4	Host Defense Peptides (magainins, esculentins, and dermaseptins), carnosine	[128]
Palm weevil larvae (*Rhynchophorus* spp.)	55–65	300–550	8–12	20–35	n-3: ~0.5-2% (ALA)<br>n-6: ~20-35% (LA)<br>n-9: ~20-40% (OA)	1–4	K, P, Mg, Fe	10–25	Chitin	[129]
Crayfish (freshwater)	75–80	80–110	15–20	1–3	n-3: ~15-30% (ALA)<br>n-6: ~5-15% (LA)	1–2	Ca, P, Zn, Se	2–6	Tropomyosin, astaxanthin	[130]

Leather	15	330	79.0	3.0	n-9: ~20-35% (OA)<br>traces	3.0	Ca, Na	traces	Keratin, tannins	[131,132]
Food animals										
Chicken Leg DW	74.0	170	15.0	11.0	n-3: ~1-2 (ALA)<br>n-6: ~18-22% (LA)<br>n-9: ~40-45% (OA)	1.0	K, P, Zn, Se, Fe	traces	Carnosine, anserine, taurine, and creatine	[133]
Common Carp (*Cyprinus carpio*) FW	76.0	130	18.0	5.0	n-3: ~10-15 (ALA)<br>n-6: ~15-20% (LA)<br>n-9: ~45-50% (OA)	1.5	P, K, Se, Mg, Zn	traces	Parvalbumin	[134,135]
