# Supplementary material for: Famine Foods: A Structured Narrative Review of Nutritional, Toxicological, Ecological, and Socio-Cultural Perspectives
Source: Foods. 2026 Oct 10;15(20):3602. doi: 10.3390/foods15203602 (PMC13703261; doi:10.3390/foods15203602)
Supplement: Supplementary file 1 [file foods-15-03602-s001.zip › Supplementary Table S3.pdf]

Supplementary Table S3. Toxic and anti-nutritional compounds in famine foods, effects on health and mitigation treatments

Famine food / vegetal or animal source	Toxic/antinutritional compound	Health effect(s)	Typical mitigation	Reference
Vegetal				
Bitter cassava (*Manihot esculenta*)	Cyanogenic glycosides (linamarin, lotaustralin)	Acute cyanide poisoning, konzo, neuropathy, thyroid dysfunction	Fermentation, grating, drying, soaking	[36,138]
Acorns (*Quercus* spp.)	Hydrolysable tannins	GI irritation, reduced protein digestibility, mineral chelation	Leaching in water, roasting	[139]
Grass pea (*Lathyrus sativus*)	β-ODAP (β-N-oxalyl-L-α,β-diaminopropionic acid)	Neurolathyrism (irreversible spastic paralysis)	Soaking, boiling, mixed diet	[140]
Bracken fern (*Pteridium aquilinum*)	Ptaquiloside	Carcinogenicity, bone marrow suppression	Repeated boiling, discarding water	[141]
Iceland moss (*Cetraria islandica*)	Lichen acids (usnic acid)	Hepatotoxicity (high doses), GI irritation	Boiling, alkali soaking	[142]
Wild yams (*Dioscorea* spp.)	Dioscorine, alkaloids	Neurotoxicity, vomiting, convulsions	Prolonged soaking/cooking	[143]
Nettles (*Urtica dioica*)	Histamine, formic acid, serotonin (trichomes)	Dermal irritation, burning pain	Cooking/blanching	[144]
Chenopodium spp.	Oxalates, saponins, nitrates	Kidney stone risk, mineral malabsorption	Boiling, discarding water	[145]
Purslane (*Portulaca oleracea*)	Oxalates	Calcium binding, nephrolithiasis risk	Boiling, discarding water or Cooking	[121]
Seaweeds (brown algae)	Excess iodine, arsenosugars (some spp.)	Thyroid dysfunction, chronic toxicity	Controlled intake, blanching	[146]
Cycad seeds	Cycasin, BMAA	Neurotoxicity, carcinogenicity	Extensive washing/fermentation	[147]
Animal				
Locusts/grasshoppers	Purines, possible pesticide accumulation	Hyperuricemia, toxin exposure	Roasting, sourcing control	[80]
Termites/insects	Chitin	Reduced digestibility, GI discomfort	Cooking, grinding	[123]

Beetle larvae	Heat-stable allergenic proteins	Allergic reactions	Cooking (partial reduction)	[148]
Freshwater snails	Trematode parasites (*Fasciola, Paragonimus*)	Hepatic/lung infection	Thorough cooking	[149]
Frogs/amphibians	Skin alkaloids (some spp.), parasites	Neurotoxicity, GI illness	Skin removal, cooking	[150]
Rodents	Leptospira, hantavirus, Salmonella	Severe zoonotic disease	Full cooking, sanitation, and hygienic conditions	[151]
Reptiles	*Salmonella* spp.	Gastroenteritis, sepsis risk	Thorough cooking under hygienic conditions	[152]
Mollusks	Paralytic shellfish toxins, domoic acid	Paralysis, seizures, death	Avoid toxic bloom harvests	[153]
Fish (starvation harvests)	Histamine (poor preservation)	Scombroid poisoning	Rapid preservation of hygienic conditions	[154]
